# Supplementary material for: Malagasy Conostigmus (Hymenoptera: Ceraphronoidea) and the secret of scutes
Source: PeerJ. 2016 Dec 13;4:e2682. doi: 10.7717/peerj.2682 (PMC5157207; doi:10.7717/peerj.2682)

Figure S1. Relationship between median cell length and number of cells as box-splot. Cell length is the longest diameter of scutes. Number of cells refers to the number of scutes/cells of a standard sized rectangular area.

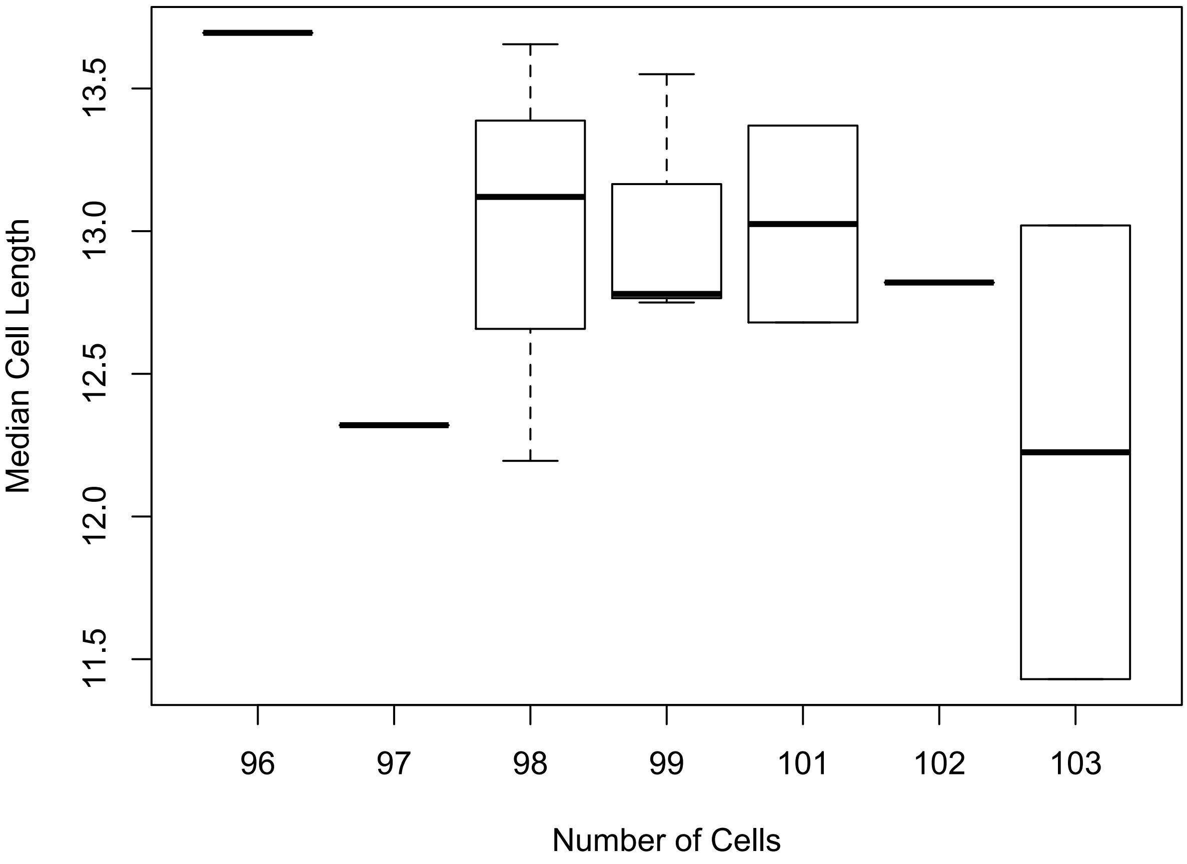

Supplement: Supplemental Information 1 — Cell length is the longest diameter of scutes. Number of cells refers to the number of scutes/cells of a standard sized rectangular area. [file peerj-04-2682-s001.pdf]
