# Supplementary material for: Malagasy Conostigmus (Hymenoptera: Ceraphronoidea) and the secret of scutes
Source: PeerJ. 2016 Dec 13;4:e2682. doi: 10.7717/peerj.2682 (PMC5157207; doi:10.7717/peerj.2682)

Figure S2. Relationship between median cell length and number of cells on the frons as linear regression. Cell length is the longest diameter of scutes. Number of cells refers to the number of scutes of a standard sized rectangular area.

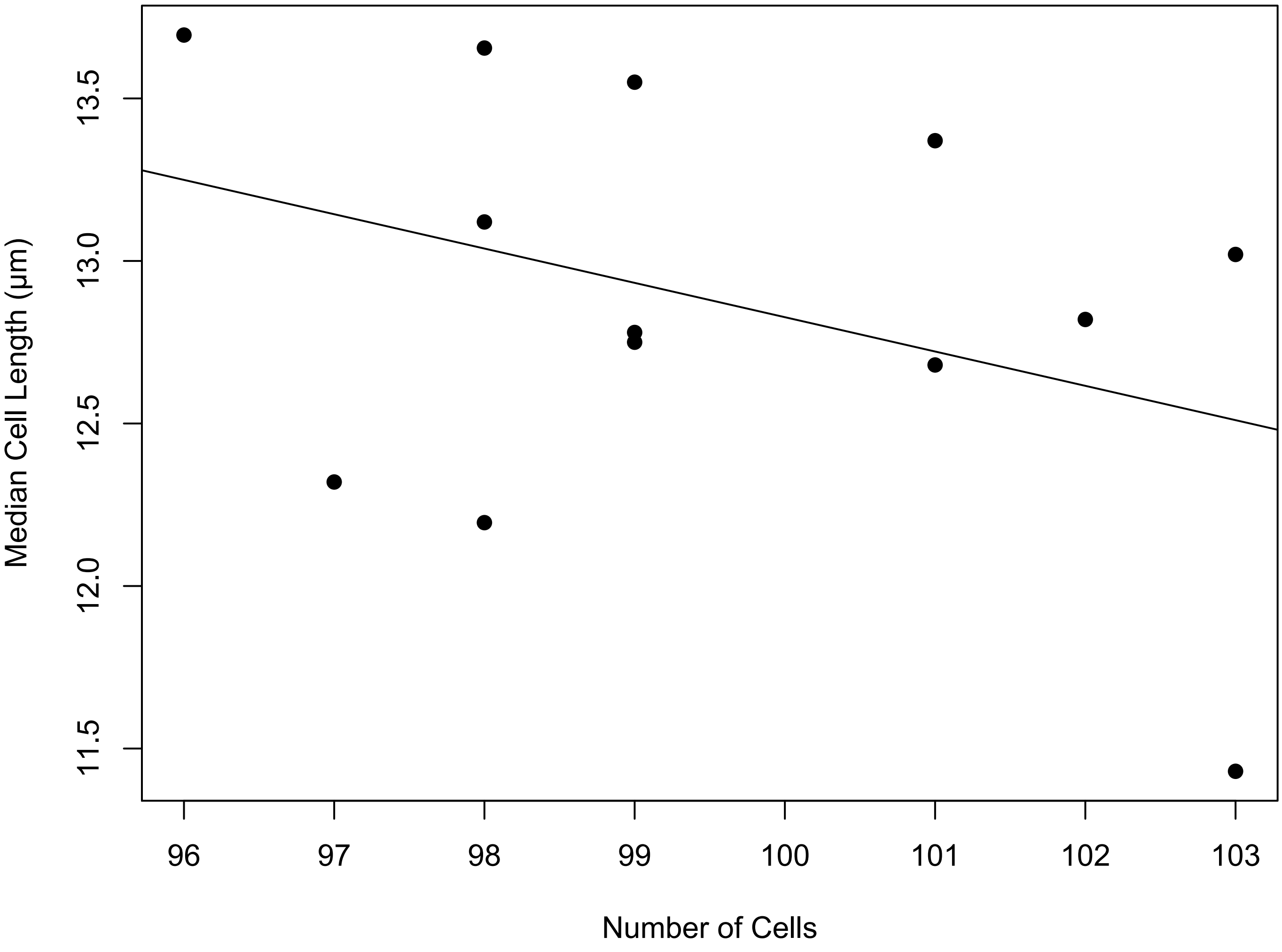

Supplement: Supplemental Information 2 — Cell length is the longest diameter of scutes. Number of cells refers to the number of scutes of a standard sized rectangular area. [file peerj-04-2682-s002.pdf]
