# Supplementary material for: Malagasy Conostigmus (Hymenoptera: Ceraphronoidea) and the secret of scutes
Source: PeerJ. 2016 Dec 13;4:e2682. doi: 10.7717/peerj.2682 (PMC5157207; doi:10.7717/peerj.2682)

Figure S3. Relationship between median cell length and number of cells on the mseonotum as linear regression. Median cell length refers to the maximum diameter of scutes. Number of cells refers to the number of scutes of a standard sized rectangular area.

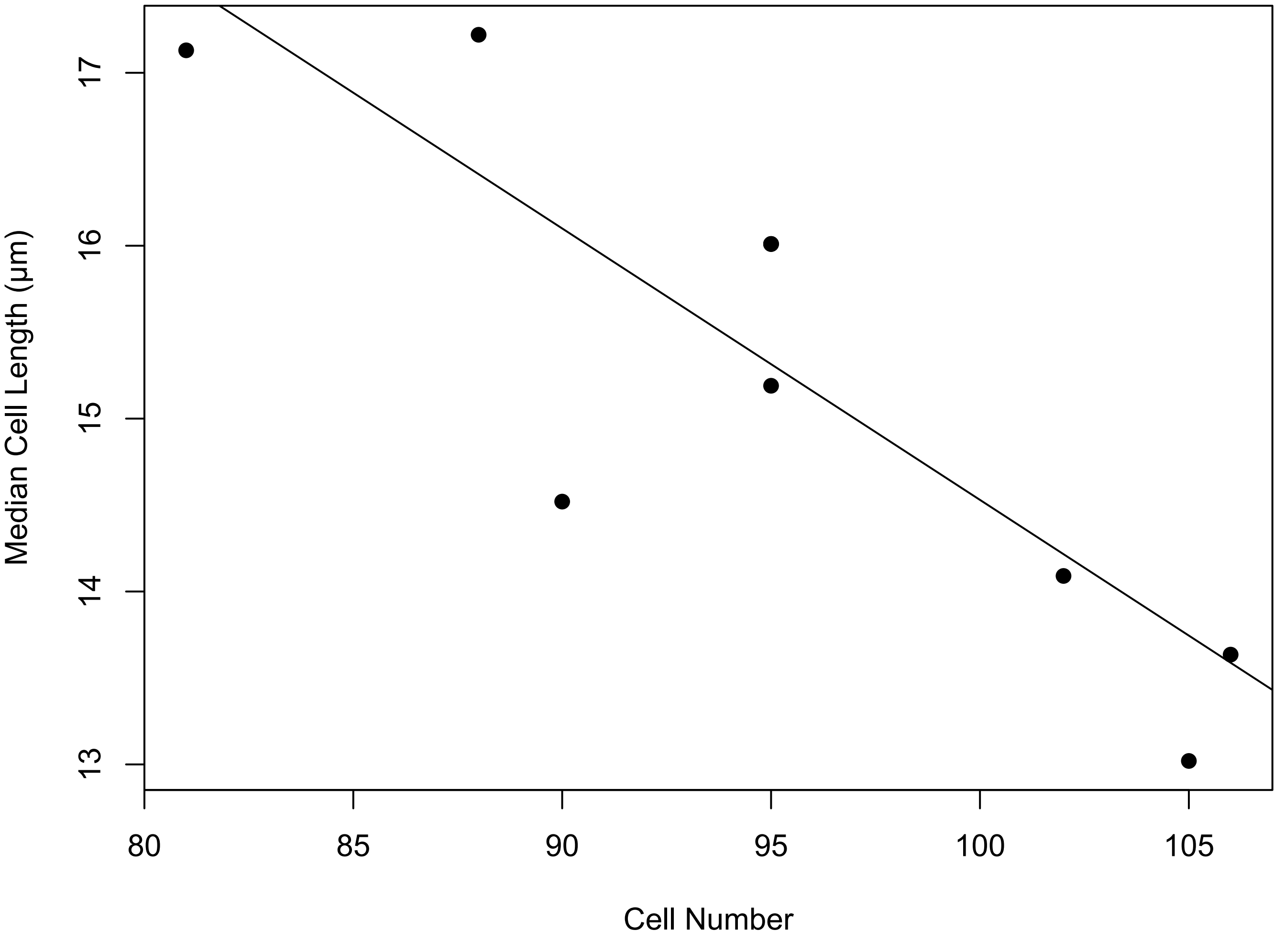

Supplement: Supplemental Information 3 — Median cell length refers to the maximum diameter of scutes. Number of cells refers to the number of scutes of a standard sized rectangular area. [file peerj-04-2682-s003.pdf]
