# Supplementary material for: Malagasy Conostigmus (Hymenoptera: Ceraphronoidea) and the secret of scutes
Source: PeerJ. 2016 Dec 13;4:e2682. doi: 10.7717/peerj.2682 (PMC5157207; doi:10.7717/peerj.2682)

Figure S4. Relationship between body size and median cell length as linear regression. Interorbital space length (IOS), measured in µm, stands as a proxy for body size. Number of cells refers to the number of scutes/cells of a standard sized rectangular area.

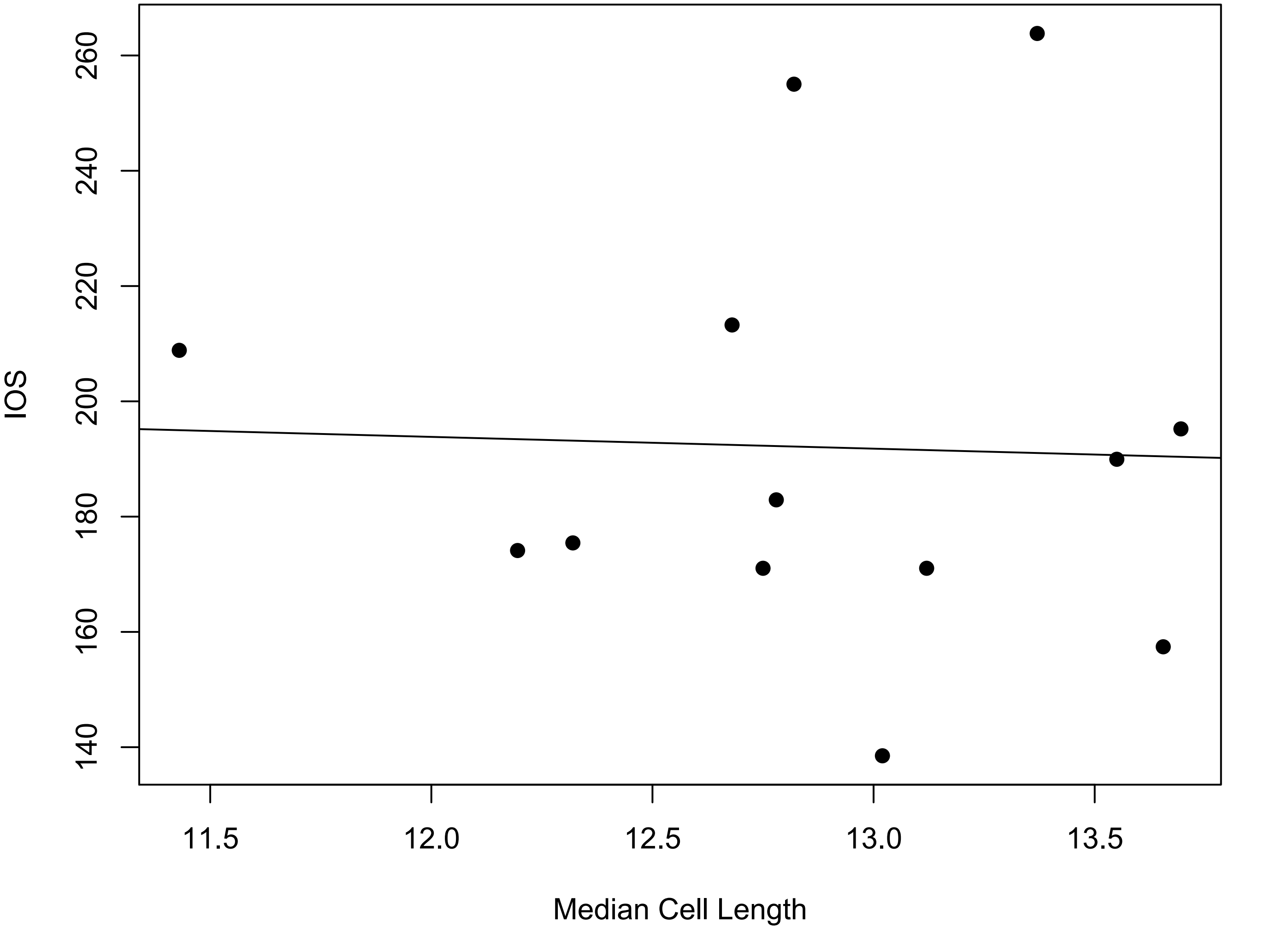

Supplement: Supplemental Information 4 — Interorbital space length (IOS), measured in μm, stands as a proxy for body size. Number of cells refers to the number of scutes/cells of a standard sized rectangular area. [file peerj-04-2682-s004.pdf]
