# Supplementary material for: Malagasy Conostigmus (Hymenoptera: Ceraphronoidea) and the secret of scutes
Source: PeerJ. 2016 Dec 13;4:e2682. doi: 10.7717/peerj.2682 (PMC5157207; doi:10.7717/peerj.2682)
Supplement: Supplemental Information 5 [file peerj-04-2682-s005.pdf]

Table S1: Verbose specimens examined

Conostigmus babaiax Dessart , 1997

Identifier(s)	Repository	Sex	Collecting event
PSUC_FEM 000006723	MRAC	female	COLL. MUS. Congo Madagascar: Mandraka II-1944 A. Seyrig HOLOTYPUS Holotype Prep. micros-copique n 9508/051

Conostigmus ballescoracas Dessart, 1997

Identifier(s)	Repository	Sex	Collecting event
CASENT 2001391; PSUC_FEM 79705	CAS	female	MADAGASCAR: Province d'Antsiranana Reserve Speciale de l'Ankarana, 22.9 km 224° SW Anivorano Nord 10-16 Feb 2001 12°54'32"S, 49°6'35"E coll. Fisher, Griswold et al. Calif. Academy of Sciences pitfall trap tropical dry forest 80m coll. code BLF2854
CASENT 2016542	CAS	female	MADAGASCAR: Toliara Province Parc Nat. d' Andohahela, Foret d'Ambohibory, 1.7 km 61° ENE Tsimelahy, 36.1 km 308° NW Tolagnaro. 16-20 I 2002. 24°55'48.0"S 46°38'44.0"E. coll. Fisher, Griswold et al. California Academy of sciences. pitfall trap- in tropical dry forest. elev. 300m. BLF4915
PSUC_FEM 8883	MRAC	female	Congo Belge : P.N.A 7-XIII-1953 H. Synave 6853 Massif Ruwenzori Mont Ngulingo pres Nyamgaleke, 2.500m, ex P.N.A HOLOTYPE Prep. micros-copique n 9507/241

Conostigmus bucephalus (Madsp. 15)

Identifier(s)	Repository	Sex	Collecting event
CASENT 2053589	CAS	female	MADAGASCAR: Province Fianarantsoa,Parc National Ranomafana, radio tower at forest edge, elev 1130m 20 March- 3 April 2003 21°15.05'S, 47°24.43'E collector: R. Harin'Hala California Acad of Sciences malaise, mixed tropical forest MA-02-09B-56

Conostigmus clavatus (Madsp. 12)

Identifier(s)	Repository	Sex	Collecting event
CASENT 2053642; PSUC_FEM 000079696	CAS	male	Madagascar: Province Fianarantsoa, Parc National Ranomafana, Belle Vue at Talatakely. Elev. 1020m. 28 Nov-6 Dec 2001 21°15.99' S, 047º25.21' E. Collector: R. Hanin'Hala California Academy of Sciences Malaise, second tropical forest. MA-02-09C-05

CASENT 2032775; PSUC_FEM 79698	CAS	male	Madagascar: Province Fianarantsoa, Parc National Ranomafana, Belle Vue at Talatakely. Elev. 1020m. 24 July -- 4 August 2002. 21°15.99' S, 47° 25.21' E Collector: R. Harin'Hala California Academy of Sciences Malaise, secondary tropical forest. MA-02-09C-36
CASENT 2046179; PSUC_FEM 79700	CAS	male	Madagascar: Prov. Fianarantsoa Parc National Ranomafana: Belle Vue at Talatakely; 1020m; -21.2655, 47.4202; 22-28.xi.2001; Malaise trap in secondary tropical forest. Collector: R. Hanin'Hala. MA-02-09C-04
CASENT 2045085; PSUC_FEM 79699	CAS	male	MADAGASCAR: Province Fianarantsoa,Parc National Ranomafana, radio tower at forest edge, 1130m 14-21 January 2002 21°15.05'S, 47°24.43'E collector: R. Harin'Hala California Acad of Sciences malaise, mixed tropical forest MA-02-09B-12
CASENT 2044514; PSUC_FEM 79703	CAS	male	MADAGASCAR: Province Fianarantsoa, Parc National Ranomafana, Vohiparara at broken bridge, el 1110 m 22-28 November 2001 21°13.57'S, 47°22.19'E collector: R. Harin'Hala California Acad of Sciences malaise trap in high altitude rainforest, MA-02-09A-04
CASENT 2045755; PSUC_FEM 79764	MRAC	male	Madagascar: Province Fianarantsoa, Parc National Ranomafana, Belle Vue at Talatakely. Elev. 1020m. 15--22 November 2001. 21°15.99' S, 47° 25.21' E Collector: R. Harin'Hala California Academy of Sciences Malaise, secondary tropical forest. MA-02-09C-03
CASENT 2044150; PSUC_FEM 000079755	CAS	male	Madagascar: Province Fianarantsoa, Parc National Ranomafana, Vohiparara at broken bridge, el 1110m 21-28 January 2002 21º13.57'S 47º22.19'E collector R.Harin'Hala California Acad of Sciences malaise trap in high altitude rainforest, MA-02-09A-13
CASENT 2046178; PSUC_FEM 79704	CAS	male	Madagascar: Prov. Fianarantsoa Parc National Ranomafana: Belle Vue at Talatakely; 1020m; -21.2655, 47.4202; 22-28.xi.2001; Malaise trap in secondary tropical forest. Collector: R. Hanin'Hala. MA-02-09C-04
CASENT 2045602	MRAC	female	MADAGASCAR: Province Fianarantsoa, Parc National Ranomafana, radio tower at forest edge; 21º15.05'S, 47º24.43'E; 1130m; 15-21 December 2001; collector R. Harin'Hala malaise, mixed tropical forest. MA-02-09B-07.
CASENT 2002179	CAS	female	MADAGASCAR: Procince d'Antananarivo 3 km 41ºNE Andranomay, 11.5km 147º SSE Anjozorobe elev 1300m, 5-13.Dec. 2000 18º28'24"S 47º57'36"E coll. Fisher,

			Griswold et al. California Acad. of Sciences montane rainforest, Malaise trap, coll. code: BLF2375
CASENT 2045509	CAS	female	MADAGASCAR: Fianarantsoa Parc National de Ranomafana Vatoharanana River 4.1 km 231º SW Ranomafana elev. 1100m 27-31Mar 2003 21º 17'24"S 047º 26'00" E California Academy of Sciences coll. Fisher, Griswold et. a. yellow pan trap, montane rainforest code: BLF8399
CASENT 2046024	CAS	female	Madagascar: Province Fianarantsoa, Parc National Ranomafana, Belle Vue at Talatakely, elev 1020m 12-19 February 2002 21°15.99'S, 47°25.21'E collector: R. Harin'Hala California Acad of Sciences Malaise, secondary tropical forest MA-02-09C-16

Conostigmus fianarantsoaensis (Mad sp3.)

Identifier(s)	Repository	Sex	Collecting event
PSUC_FEM 000079738; CASENT 2053304	CAS	male	MADAGASCAR: Province Fianarantsoa, Parc National Ranomafana, Vohiparara, at broken bridge, el 1110m 25 July- 3 August 2002 21°13.57'S, 47°22.19'E collector: R. Harin'Hala California Acad of Sciences malaise trap in high altitude rainforest, MA-02-09A-36
IM 2288; CASENT 2032776	CAS	male	Madagascar: Province Fianarantsoa, Parc National Ranomafana, Belle Vue at Talatakely. Elev. 1020m. 24 July -- 4 August 2002. 21°15.99' S, 47° 25.21' E Collector: R. Harin'Hala California Academy of Sciences Malaise, secondary tropical forest. MA-02-09C-36
PSUC_FEM 000079695; CASENT 2032773	CAS	male	Madagascar: Province Fianarantsoa, Parc National Ranomafana, Belle Vue at Talatakely. Elev. 1020m. 24 July -- 4 August 2002. 21°15.99' S, 47° 25.21' E Collector: R. Harin'Hala California Academy of Sciences Malaise, secondary tropical forest. MA-02-09C-36
PSUC_FEM 000079762; CASENT 2053444	MRAC	male	MADAGASCAR: Province Fianarantsoa,Parc National Ranomafana, Belle Vue Talatakely, elev 1020m 28 May-6 June 2003 21°15.99'S, 47°25.21'E collector: R. Harin'Hala California Acad of Sciences malaise, secondary tropical forest MA-02-09C-62
PSUC_FEM 000079737; CASENT 2045086	CAS	male	MADAGASCAR: Province Fianarantsoa,Parc National Ranomafana, radio tower at forest edge, 1130m 14-21 January 2002 21°15.05'S, 47°24.43'E collector: R. Harin'Hala California Acad of Sciences malaise, mixed tropical forest MA-02-09B-12

PSUC_FEM 000079740; CASENT 2053588	CAS	male	MADAGASCAR: Province Fianarantsoa,Parc National Ranomafana, radio tower at forest edge, elev 1130m 20 March- 3 April 2003 21°15.05'S, 47°24.43'E collector: R. Harin'Hala California Acad of Sciences malaise, mixed tropical forest MA-02-09B-56
PSUC_FEM 000079749; CASENT 2046018	CAS	male	Madagascar: Province Fianarantsoa, Parc National Ranomafana, Belle Vue at Talatakely, elev 1020m 12-19 February 2002 21°15.99'S, 47°25.21'E collector: R. Harin'Hala California Acad of Sciences Malaise, secondary tropical forest MA-02-09C-16
PSUC_FEM 000079760; CASENT 2041944	CAS	male	Madagascar: Province Fianarantsoa, Parc National Ranomafana, Vohiparara at broken bridge, el 1110m 6-15 December 2001 21º13.57'S 47º22.19'E collector R.Harin'Hala California Acad of Sciences malaise trap in high altitude rainforest, MA-02-09A-06
CASENT 2053306	CAS		MADAGASCAR: Province Fianarantsoa, Parc National Ranomafana, Vohiparara, at broken bridge, el 1110m 25 July- 3 August 2002 21°13.57'S, 47°22.19'E collector: R. Harin'Hala California Acad of Sciences malaise trap in high altitude rainforest, MA-02-09A-36
PSUC_FEM 000079734; CASENT 2044516	CAS	male	MADAGASCAR: Province Fianarantsoa, Parc National Ranomafana, Vohiparara at broken bridge, el 1110 m 22-28 November 2001 21°13.57'S, 47°22.19'E collector: R. Harin'Hala California Acad of Sciences malaise trap in high altitude rainforest, MA-02-09A-04
CASENT 2053691	CAS	male	MADAGASCAR: Province Fianarantsoa,Ranomafana JIRAMA water works 21°14.91'S, 47°27.13'E 16 Oct-8 Nov 2001 collector: R. Harin'Hala California Acad of Sciences malaise trap near river elev 690 m MA-02-09D-01
CASENT 2022988	CAS	male	MADAGASCAR: Province Fianarantsoa, Ranomafana JIRAMA water works 21°14.91'S, 47°27.13'E 21-24 December 2001 collector: R. Harin'Hala California Acad of Sciences Malaise trap near river elev 690m MA-02-09D-08
CASENT 2046177; PSUC_FEM 79713	CAS	male	Madagascar: Prov. Fianarantsoa Parc National Ranomafana: Belle Vue at Talatakely; 1020m; -21.2655, 47.4202; 22-28.xi.2001; Malaise trap in secondary tropical forest. Collector: R. Hanin'Hala. MA-02-09C-04
CASENT 2044151; PSUC_FEM 79708	CAS	male	Madagascar: Province Fianarantsoa, Parc National Ranomafana, Vohiparara at broken bridge, el 1110m

			21-28 January 2002 21º13.57'S 47º22.19'E collector R.Harin'Hala California Acad of Sciences malaise trap in high altitude rainforest, MA-02-09A-13
CASENT 2046180; PSUC_FEM 79710	CAS	male	Madagascar: Prov. Fianarantsoa Parc National Ranomafana: Belle Vue at Talatakely; 1020m; -21.2655, 47.4202; 22-28.xi.2001; Malaise trap in secondary tropical forest. Collector: R. Hanin'Hala. MA-02-09C-04
CASENT 2045601; PSUC_FEM 79718	CAS	male	MADAGASCAR: Province Fianarantsoa, Parc National Ranomafana, radio tower at forest edge; 21º15.05'S, 47º24.43'E; 1130m; 15-21 December 2001; collector R. Harin'Hala malaise, mixed tropical forest. MA-02-09B-07.
CASENT 2053667; PSUC_FEM 79707	CAS	male	MADAGASCAR: Province Fianarantsoa, Parc National Ranomafana, radio tower at forest edge; 1130m; 22-28 Nov. 2001; 21°15.05'S, 47°24.43'E. collector: R. Harin'Hala California Academy of Sciences, malaise, mixed tropical rainforest. MT; MA-02-09B-04
CASENT 2045975	CAS	male	MADAGASCAR: Province Fianarantsoa, Parc National Ranomafana, radio tower at forest edge elev 1130m 16 Oct.-8 Nov. 2001 21°15.05’ S, 47°24.43’E Collector R. Harin’Hala California Acad of Science Malaise, mixed tropical forest MA-02-09B-01
CASENT 2045741	CAS	female	Madagascar: Province Fianarantsoa, Parc National Ranomafana, Belle Vue at Talatakely. elev 1020m 23May- 3 June 2002. 21°15.99'S, 47°25.21'E. collector: R. Harin'Hala. California Academy of Sciences. Malaise, secondary tropical forest MA-02-09C-30
CASENT 2053641	CAS	female	Madagascar: Province Fianarantsoa, Parc National Ranomafana, Belle Vue at Talatakely. Elev. 1020m. 28 Nov-6 Dec 2001 21º15.99' S, 047º25.21' E. Collector: R. Hanin'Hala California Academy of Sciences Malaise, second tropical forest. MA-02-09C-05
CASENT 2053303	CAS	female	MADAGASCAR: Province Fianarantsoa, Parc National Ranomafana, Vohiparara, at broken bridge, el 1110m 25 July- 3 August 2002 21°13.57'S, 47°22.19'E collector: R. Harin'Hala California Acad of Sciences malaise trap in high altitude rainforest, MA-02-09A-36
PSUC_FEM 000079756; CASENT 2019263	CAS	male	MADAGASCAR: Province Fianarantsoa Foret d'Tolonapina Sud I Mandriandry X-2000 21°35'30" S, 47°29'10" E colls: R. Harin'Hala & M.E. Irwin, malaise trap 750m MA-01-13-01

Conostigmus longulus Dessart, 1997

Identifier(s)	Repository	Sex	Collecting event
PSUC_FEM 000079748; CASENT 2003474	CAS	male	MADAGASCAR: Province d'Antsiranana, Parc National Montagne d' Ambre, elev 960m 26-29 Jan 2001 12°30'52" S, 49°10'53" E M.E. Irwin, E.I. Schlinger & R. Harin'Hala collectors, malaise trap MA-01-01A-02
PSUC_FEM 000079745; CASENT 2044755	CAS	male	MADAGASCAR: Province Fianarantsoa, Parc National Ranomafana, radio tower at forest edge, elev 1130m 21-28 January 2002 21°15.05' S, 47°24.43' E collector: R.Harin'Hala California Acad of Sciences malaise, mixed tropical forest MA-02-09B-13
CASENT 2046098; PSUC_FEM 79754	CAS	male	Madagascar:Province Fianarantsoa, Parc National Ranarantsoa, Belle Vue at Talatakely. elev. 1020m 10-14 January 2002. 21°15.99’ S, 47°25.21’ E Collector: R. Harin'Hala California Academy of sciences. Malaise, secondary tropical forest. MA-02-09C-11
PSUC_FEM 000079735; CASENT 2053687	CAS	male	MADAGASCAR: Province Fianarantsoa, Parc National Ranomafana, Belle Vue at Talatakely, elev 1020 m 16 Oct- 8 Nov 2001 21°15.99' S, 47°25.21' E collector: R.Harin'Hala California Acad of Sciences malaise, secondary tropical forest MA-02-09C-01
PSUC_FEM 000079732; CASENT 2044825	CAS	male	MADAGASCAR: Province Fianarantsoa, Parc National Ranomafana, radio tower at forest edge; -21.251,47.407; 1130m; 12-19.iii.2002; MT; MA-02-09B-20; R. Harin'Hala
PSUC_FEM 000079757; CASENT 2041918	CAS	male	MADAGASCAR: Province Fianarantsoa, Parc National Ranomafana, Vohiparara, at broken bridge, el 1110m 12-19 March 2002 21°13.57' S, 47°22.19' E collector: R.Harin'Hala California Acad of Sciences malaise trap in high altitude rainforest, MA-02-09A-20
CASENT 2046100	CAS	male	MADAGASCAR: Province Fianarantsoa, Parc National Ranomafana, Belle Vue at Talatakely, elev 1020m 23-28 April 2002 21°15.99'S, 47°25.21'E collector: R. Harin'Hala California Acad of Sciences Malaise, secondary tropical forest MA-02-09C-26
CASENT 2053688	CAS	male	MADAGASCAR: Province Fianarantsoa, Parc National Ranomafana, Belle Vue at Talatakely, elev 1020 m 16 Oct- 8 Nov 2001 21°15.99' S, 47°25.21' E collector: R.Harin'Hala California Acad of Sciences malaise, secondary tropical forest MA-02-09C-01

CASENT 2044193	CAS	male	Madagascar:Province Fianarantsoa, Parc National Ranomafana, Vohiparara at broken bridge. elev. 1020m 4-12 February 2002. 21°13.57’S, 47°22.19’ E Collector: R. Harin'Hala California Academy of sciences. Malaise trap in high altitude rainforest MA-02-09A-15
PSUC_FEM 000079753; CASENT 2044511	MRAC	male	Madagascar: Toamasina Province, botanic garden near entrance to Andasibe National Park, 8-16 October 2001 18º55.58'S, 48º24,47'E collector: R. Harin'Hala California Acad. of Sciences Malaise trap- tropical forest elev 1025m, MA-01-08B-15
CASENT 2053554	CAS	female	MADAGASCAR: Province Fianarantsoa, Parc National Ranomafana, radio tower at forest edge, el 1130m 9-20 March 2003 21°15.05' S, 47°24.43' E collector: R.Harin'Hala California Acad of Sciences malaise mixed tropical forest, MA-02-09B-55
CASENT 2053308	MRAC	female	MADAGASCAR: Mahajanga Province, Parc National de Namoroka, 16.9km 317°NW Vilanandro elev 100m 12-16 Nov 2002 16°24'24" S, 045°18'36" E coll. Fisher, Griswold et al. California Acad. of Sciences malaise trap-tropical dry forest, BLF6581
CASENT 2040771	CAS	female	MADAGASCAR: Toamasina Province, botanic garden near entrance to Andasibe National Park, 1-7 November 2001 18°55.58'S, 48°24.47'E collector: R. Harin'Hala California Acad of Sciences Malaise trap-tropical forest elev 1025m MA-01-08B-18
CASENT 2040900	CAS	female	Madagascar: Province d'Antananarivo 3 km 41ºNE Andranomay, 11.5 km 147º SSE Anjozorobe elev 1300m, 5-13 Dec 2000 18º28'24"S 47º57'36"E coll. Fisher, Griswold et al California Acad. of Sciences montane rainforest, malaise trap, coll. code: BLF2372
CASENT 2002193	CAS	female	MADAGASCAR: Procince d'Antananarivo 3 km 41ºNE Andranomay, 11.5km 147º SSE Anjozorobe elev 1300m, 5-13.Dec. 2000 18º28'24"S 47º57'36"E coll. Fisher, Griswold et al. California Acad. of Sciences montane rainforest, Malaise trap, coll. code: BLF2375
CASENT 2009756	CAS	female	Madagascar:Province d' Antsiranana, botanic garden near the entrance to Andasibe National Park. 1-5 Sept. 2001.18°55.58' S. 48°24.47' E. coll. R. Harin'Hala. California Academy of Sciences. malaise trap- tropical forest. elev. 1025m. MA-01-08B-11
PSUC_FEM 8919	MRAC	male	COLL. MUS. Congo Madagascar: Mandraka II-1944 A.

			Seyrig HOLOTYPUS Holotype Prep. micros-copique n 9508/051

Conostigmus lucidus (Madsp.8)

Identifier(s)	Repository	Sex	Collecting event
CASENT 2004751	CAS	female	Madagascar: Province d'Antananarivo: 3 km 41º NE Andranamay: 11.5 km 147º SSE Anjozorobe: 1300 m 18º 21' 24" S, 47º 57' 36" E 5-13.xii.2000 montane forest B. L. Fisher et al. CAS collection code: BLF2375
CASENT 2004743	CAS	female	MADAGASCAR: Procince d'Antananarivo 3 km 41ºNE Andranomay, 11.5km 147º SSE Anjozorobe elev 1300m, 5-13.Dec. 2000 18º28'24"S 47º57'36"E coll. Fisher, Griswold et al. California Acad. of Sciences montane rainforest, Malaise trap, coll. code: BLF2375
CASENT 2002181	CAS	female	MADAGASCAR: Procince d'Antananarivo 3 km 41ºNE Andranomay, 11.5km 147º SSE Anjozorobe elev 1300m, 5-13.Dec. 2000 18º28'24"S 47º57'36"E coll. Fisher, Griswold et al. California Acad. of Sciences montane rainforest, Malaise trap, coll. code: BLF2375
CASENT 2040895	MRAC	female	Madagascar: Province d'Antananarivo 3 km 41ºNE Andranomay, 11.5 km 147º SSE Anjozorobe elev 1300m, 5-13 Dec 2000 18º28'24"S 47º57'36"E coll. Fisher, Griswold et al California Acad. of Sciences montane rainforest, malaise trap, coll. code: BLF2372
CASENT 2046026	CAS	female	Madagascar: Province Fianarantsoa. Parc National Ranomafana: Belle Vue at Talatakely. Elev. 1020m 14-21 January 2002. 21º15.99'S, 47º25.21'E collector: R. Harin'Hala California Acad. of Sciences Malaise, secondary tropical forest. MA-02-09C-12
CASENT 2045754	CAS	female	Madagascar: Province Fianarantsoa, Parc National Ranomafana, Belle Vue at Talatakely. Elev. 1020m. 15--22 November 2001. 21°15.99' S, 47° 25.21' E Collector: R. Harin'Hala California Academy of Sciences Malaise, secondary tropical forest. MA-02-09C-03
CASENT 2001309; PSUC_FEM 79752	CAS	male	MADAGASCAR: Province d'Antananarivo 3km 41° NE Andranomay, 11.5km 147°SSE Anjozobe 1300m 3-13 Dec 2000 18°28'24" S. 47°57'36" E coll. Fisher, Griswold et al. California Acad of Sciences sifted litter in montane rainforest BLF2464
CASENT 2046176;	CAS	male	Madagascar: Prov. Fianarantsoa Parc National

PSUC_FEM 79743			Ranomafana: Belle Vue at Talatakely; 1020m; -21.2655, 47.4202; 22-28.xi.2001; Malaise trap in secondary tropical forest. Collector: R. Hanin'Hala. MA-02-09C-04

Conostigmus macrocupula (Mad sp.5)

Identifier(s)	Repository	Sex	Collecting event
CASENT 2046181	CAS	male	Madagascar: Prov. Fianarantsoa Parc National Ranomafana: Belle Vue at Talatakely; 1020m; -21.2655, 47.4202; 22-28.xi.2001; Malaise trap in secondary tropical forest. Collector: R. Hanin'Hala. MA-02-09C-04
CASENT 2053451	CAS	male	MADAGASCAR: Province Fianarantsoa, Parc National Ranomafana, Belle Vue at Talatakely, elev 1020m 21 March-12 April 2003 21°15.99'S, 47°25.21'E collector: R. Harin'Hala California Acad of Sciences malaise, secondary tropical forest MA-02-09C-57
PSUC_FEM 000079750; CASENT 2053442	CAS	male	MADAGASCAR: Province Fianarantsoa,Parc National Ranomafana, Belle Vue Talatakely, elev 1020m 28 May-6 June 2003 21°15.99'S, 47°25.21'E collector: R. Harin'Hala California Acad of Sciences malaise, secondary tropical forest MA-02-09C-62
CASENT 2046023	CAS	male	Madagascar: Province Fianarantsoa, Parc National Ranomafana, Belle Vue at Talatakely, elev 1020m 12-19 February 2002 21°15.99'S, 47°25.21'E collector: R. Harin'Hala California Acad of Sciences Malaise, secondary tropical forest MA-02-09C-16
CASENT 2046025	CAS	male	Madagascar: Province Fianarantsoa, Parc National Ranomafana, Belle Vue at Talatakely, elev 1020m 12-19 February 2002 21°15.99'S, 47°25.21'E collector: R. Harin'Hala California Acad of Sciences Malaise, secondary tropical forest MA-02-09C-16
CASENT 2046022	MRAC	male	Madagascar: Province Fianarantsoa, Parc National Ranomafana, Belle Vue at Talatakely, elev 1020m 12-19 February 2002 21°15.99'S, 47°25.21'E collector: R. Harin'Hala California Acad of Sciences Malaise, secondary tropical forest MA-02-09C-16
PSUC_FEM 000079742; CASENT 2053572	CAS	male	MADAGASCAR: Province Fianarantsoa, Parc National Ranomafana, Belle Vue at Talatakely, elev 1020m 4-12 February 2002 21°15.99'S, 47°25.21'E collector: R. Harin'Hala California Acad of Sciences malaise, secondary tropical forest MA-02-09C-15

PSUC_FEM 000079741; CASENT 2044710	CAS	male	Madagascar: Province Fianarantsoa. Parc National d'lsalo 9.1km 354° N Ranohira. elev. 725m. 27-31 January 2003. 22°28'54" S, 045°27'42" E collector: Fisher, Griswold et al. California Acad. of Sciences Malaise trap in gallery forest collection code: BLF7303

Conostigmus madagascariensis (MCsp1)

Identifier(s)	Repository	Sex	Collecting event
CASENT 2041942	CAS	male	Madagascar: Province Fianarantsoa, Parc National Ranomafana, Vohiparara at broken bridge, el 1110m 6-15 December 2001 21º13.57'S 47º22.19'E collector R.Harin'Hala California Acad of Sciences malaise trap in high altitude rainforest, MA-02-09A-06
CASENT 2040907	CAS	male	Madagascar: Province d'Antananarivo 3 km 41ºNE Andranomay, 11.5 km 147º SSE Anjozorobe elev 1300m, 5-13 Dec 2000 18º28'24"S 47º57'36"E coll. Fisher, Griswold et al California Acad. of Sciences montane rainforest, malaise trap, coll. code: BLF2372
CASENT 2004750	CAS	male	MADAGASCAR: Procince d'Antananarivo 3 km 41ºNE Andranomay, 11.5km 147º SSE Anjozorobe elev 1300m, 5-13.Dec. 2000 18º28'24"S 47º57'36"E coll. Fisher, Griswold et al. California Acad. of Sciences montane rainforest, Malaise trap, coll. code: BLF2375
CASENT 2004754	CAS	male	MADAGASCAR: Procince d'Antananarivo 3 km 41ºNE Andranomay, 11.5km 147º SSE Anjozorobe elev 1300m, 5-13.Dec. 2000 18º28'24"S 47º57'36"E coll. Fisher, Griswold et al. California Acad. of Sciences montane rainforest, Malaise trap, coll. code: BLF2375
CASENT 2002178	CAS	male	MADAGASCAR: Procince d'Antananarivo 3 km 41ºNE Andranomay, 11.5km 147º SSE Anjozorobe elev 1300m, 5-13.Dec. 2000 18º28'24"S 47º57'36"E coll. Fisher, Griswold et al. California Acad. of Sciences montane rainforest, Malaise trap, coll. code: BLF2375
CASENT 2002191	CAS	male	MADAGASCAR: Procince d'Antananarivo 3 km 41ºNE Andranomay, 11.5km 147º SSE Anjozorobe elev 1300m, 5-13.Dec. 2000 18º28'24"S 47º57'36"E coll. Fisher, Griswold et al. California Acad. of Sciences montane rainforest, Malaise trap, coll. code: BLF2375
CASENT 2002187	CAS	male	MADAGASCAR: Procince d'Antananarivo 3 km 41ºNE Andranomay, 11.5km 147º SSE Anjozorobe elev 1300m, 5-13.Dec. 2000 18º28'24"S 47º57'36"E coll. Fisher,

			Griswold et al. California Acad. of Sciences montane rainforest, Malaise trap, coll. code: BLF2375
CASENT 2004746	CAS	male	MADAGASCAR: Procince d'Antananarivo 3 km 41ºNE Andranomay, 11.5km 147º SSE Anjozorobe elev 1300m, 5-13.Dec. 2000 18º28'24"S 47º57'36"E coll. Fisher, Griswold et al. California Acad. of Sciences montane rainforest, Malaise trap, coll. code: BLF2375
CASENT 2004748	CAS	male	MADAGASCAR: Procince d'Antananarivo 3 km 41ºNE Andranomay, 11.5km 147º SSE Anjozorobe elev 1300m, 5-13.Dec. 2000 18º28'24"S 47º57'36"E coll. Fisher, Griswold et al. California Acad. of Sciences montane rainforest, Malaise trap, coll. code: BLF2375
CASENT 2041648	CAS	female	Madagascar: Toamasina Province, botanic garden near entrance to Andasibe National Park, 16-23 November 2001 18º55.58'S, 48º24.47'E Collector: R. Harin'Hala California Acad. of Sciences Malaise trap- tropical forest elev 1025m, MA-01-08B-20
CASENT 2040892	CAS	male	Madagascar: Province d'Antananarivo 3 km 41ºNE Andranomay, 11.5 km 147º SSE Anjozorobe elev 1300m, 5-13 Dec 2000 18º28'24"S 47º57'36"E coll. Fisher, Griswold et al California Acad. of Sciences montane rainforest, malaise trap, coll. code: BLF2372
CASENT 2040901	CAS	male	Madagascar: Province d'Antananarivo 3 km 41ºNE Andranomay, 11.5 km 147º SSE Anjozorobe elev 1300m, 5-13 Dec 2000 18º28'24"S 47º57'36"E coll. Fisher, Griswold et al California Acad. of Sciences montane rainforest, malaise trap, coll. code: BLF2372
CASENT 2004744	CAS	male	MADAGASCAR: Procince d'Antananarivo 3 km 41ºNE Andranomay, 11.5km 147º SSE Anjozorobe elev 1300m, 5-13.Dec. 2000 18º28'24"S 47º57'36"E coll. Fisher, Griswold et al. California Acad. of Sciences montane rainforest, Malaise trap, coll. code: BLF2375
CASENT 2044507	CAS	male	Madagascar: Toamasina Province, botanic garden near entrance to Andasibe National Park, 8-16 October 2001 18º55.58'S, 48º24,47'E collector: R. Harin'Hala California Acad. of Sciences Malaise trap- tropical forest elev 1025m, MA-01-08B-15
CASENT 2002190	CAS	male	MADAGASCAR: Procince d'Antananarivo 3 km 41ºNE Andranomay, 11.5km 147º SSE Anjozorobe elev 1300m, 5-13.Dec. 2000 18º28'24"S 47º57'36"E coll. Fisher, Griswold et al. California Acad. of Sciences montane

			rainforest, Malaise trap, coll. code: BLF2375
CASENT 2040899	CAS	male	Madagascar: Province d'Antananarivo 3 km 41ºNE Andranomay, 11.5 km 147º SSE Anjozorobe elev 1300m, 5-13 Dec 2000 18º28'24"S 47º57'36"E coll. Fisher, Griswold et al California Acad. of Sciences montane rainforest, malaise trap, coll. code: BLF2372
CASENT 2040896	CAS	male	Madagascar: Province d'Antananarivo 3 km 41ºNE Andranomay, 11.5 km 147º SSE Anjozorobe elev 1300m, 5-13 Dec 2000 18º28'24"S 47º57'36"E coll. Fisher, Griswold et al California Acad. of Sciences montane rainforest, malaise trap, coll. code: BLF2372
CASENT 2040897	CAS	male	Madagascar: Province d'Antananarivo 3 km 41ºNE Andranomay, 11.5 km 147º SSE Anjozorobe elev 1300m, 5-13 Dec 2000 18º28'24"S 47º57'36"E coll. Fisher, Griswold et al California Acad. of Sciences montane rainforest, malaise trap, coll. code: BLF2372
CASENT 2045756	CAS	male	Madagascar: Province Fianarantsoa, Parc National Ranomafana, Belle Vue at Talatakely. Elev. 1020m. 15--22 November 2001. 21°15.99' S, 47° 25.21' E Collector: R. Harin'Hala California Academy of Sciences Malaise, secondary tropical forest. MA-02-09C-03
CASENT 2040906	CAS	male	Madagascar: Province d'Antananarivo 3 km 41ºNE Andranomay, 11.5 km 147º SSE Anjozorobe elev 1300m, 5-13 Dec 2000 18º28'24"S 47º57'36"E coll. Fisher, Griswold et al California Acad. of Sciences montane rainforest, malaise trap, coll. code: BLF2372
CASENT 2004742	CAS	male	MADAGASCAR: Procince d'Antananarivo 3 km 41ºNE Andranomay, 11.5km 147º SSE Anjozorobe elev 1300m, 5-13.Dec. 2000 18º28'24"S 47º57'36"E coll. Fisher, Griswold et al. California Acad. of Sciences montane rainforest, Malaise trap, coll. code: BLF2375
CASENT 2046020	CAS	male	Madagascar: Province Fianarantsoa, Parc National Ranomafana, Belle Vue at Talatakely, elev 1020m 12-19 February 2002 21°15.99'S, 47°25.21'E collector: R. Harin'Hala California Acad of Sciences Malaise, secondary tropical forest MA-02-09C-16
CASENT 2040891	CAS	male	Madagascar: Province d'Antananarivo 3 km 41ºNE Andranomay, 11.5 km 147º SSE Anjozorobe elev 1300m, 5-13 Dec 2000 18º28'24"S 47º57'36"E coll. Fisher, Griswold et al California Acad. of Sciences montane rainforest, malaise trap, coll. code: BLF2372

CASENT 2002189	MRAC	male	MADAGASCAR: Procince d'Antananarivo 3 km 41ºNE Andranomay, 11.5km 147º SSE Anjozorobe elev 1300m, 5-13.Dec. 2000 18º28'24"S 47º57'36"E coll. Fisher, Griswold et al. California Acad. of Sciences montane rainforest, Malaise trap, coll. code: BLF2375
CASENT 2002188	CAS	male	MADAGASCAR: Procince d'Antananarivo 3 km 41ºNE Andranomay, 11.5km 147º SSE Anjozorobe elev 1300m, 5-13.Dec. 2000 18º28'24"S 47º57'36"E coll. Fisher, Griswold et al. California Acad. of Sciences montane rainforest, Malaise trap, coll. code: BLF2375
CASENT 2040894	CAS	male	Madagascar: Province d'Antananarivo 3 km 41ºNE Andranomay, 11.5 km 147º SSE Anjozorobe elev 1300m, 5-13 Dec 2000 18º28'24"S 47º57'36"E coll. Fisher, Griswold et al California Acad. of Sciences montane rainforest, malaise trap, coll. code: BLF2372
CASENT 2053503	CAS	male	MADAGASCAR: Province Fianarantsoa, Ranomafana JIRAMA water works 21°14.19'S, 47°27.13'E 2-10 January 2002 collector: R. Harin'Hala California Acad of Sciences Malaise trap near river elev 690m MA-02-09D-10
CASENT 2044912	CAS	male	MADAGASCAR: Province Fianarantsoa, Parc National Ranomafana, radio tower at forest edge, elev 1130m 12-19 February 2002 21°15.05'S, 47°24.43E collector: R. Harin'Hala California Acad of Sciences Malaise mixed tropical forest MA-02-09B-16
CASENT 2044913	CAS	male	MADAGASCAR: Province Fianarantsoa, Parc National Ranomafana, radio tower at forest edge, elev 1130m 12-19 February 2002 21°15.05'S, 47°24.43E collector: R. Harin'Hala California Acad of Sciences Malaise mixed tropical forest MA-02-09B-16
CASENT 2040893	CAS	male	Madagascar: Province d'Antananarivo: 3 km 41º NE Andranamay: 11.5 km 147º SSE Anjozorobe: 1300 m 18º 21' 24" S, 47º 57' 36" E 5-13.xii.2000 montane forest B. L. Fisher et al. CAS collection code: BLF2375
PSUC_FEM 000079763; CASENT 2045250	CAS	male	MADAGASCAR: Province Fianarantsoa, Ranomafana JIRAMA water works 21°14.19'S, 47°27.13'E 10-14 January 2002 collector: R. Harin'Hala California Acad of Sciences Malaise trap near river elev 690m MA-02-09D-11
PSUC_FEM 000079702; CASENT	CAS	male	MADAGASCAR: Procince d'Antananarivo 3 km 41ºNE Andranomay, 11.5km 147º SSE Anjozorobe elev 1300m,

2002192			5-13.Dec. 2000 18º28'24"S 47º57'36"E coll. Fisher, Griswold et al. California Acad. of Sciences montane rainforest, Malaise trap, coll. code: BLF2375
IM 2289; CASENT 2040902	CAS	male	Madagascar: Province d'Antananarivo 3 km 41ºNE Andranomay, 11.5 km 147º SSE Anjozorobe elev 1300m, 5-13 Dec 2000 18º28'24"S 47º57'36"E coll. Fisher, Griswold et al California Acad. of Sciences montane rainforest, malaise trap, coll. code: BLF2372
PSUC_FEM 000079761; CASENT 2041820	CAS	male	MADAGASCAR: Toamasina Province, botanic garden near entrance to Andasibe National Park 6-16 November 2001 18°55.58'S, 48°24.47'E collector: R. Harin'Hala California Acad of Sciences Malaise trap-tropical forest elev 1025m, MA-01-08B-19
PSUC_FEM 000079759; CASENT 2004745	CAS	male	MADAGASCAR: Procince d'Antananarivo 3 km 41ºNE Andranomay, 11.5km 147º SSE Anjozorobe elev 1300m, 5-13.Dec. 2000 18º28'24"S 47º57'36"E coll. Fisher, Griswold et al. California Acad. of Sciences montane rainforest, Malaise trap, coll. code: BLF2375
CASENT 2040908; PSUC_FEM 79697	CAS	male	Madagascar: Province d'Antananarivo 3 km 41ºNE Andranomay, 11.5 km 147º SSE Anjozorobe elev 1300m, 5-13 Dec 2000 18º28'24"S 47º57'36"E coll. Fisher, Griswold et al California Acad. of Sciences montane rainforest, malaise trap, coll. code: BLF2372
PSUC_FEM PSUC_79714; CASENT 2041941	CAS	male	Madagascar: Province Fianarantsoa, Parc National Ranomafana, Vohiparara at broken bridge, el 1110m 6-15 December 2001 21º13.57'S 47º22.19'E collector R.Harin'Hala California Acad of Sciences malaise trap in high altitude rainforest, MA-02-09A-06
CASENT 2022987	CAS	male	MADAGASCAR: Province Fianarantsoa, Ranomafana JIRAMA water works 21°14.91'S, 47°27.13'E 21-24 December 2001 collector: R. Harin'Hala California Acad of Sciences Malaise trap near river elev 690m MA-02-09D-08
CASENT 2041940; PSUC_FEM 79716	CAS	male	Madagascar: Province Fianarantsoa, Parc National Ranomafana, Vohiparara at broken bridge, el 1110m 6-15 December 2001 21º13.57'S 47º22.19'E collector R.Harin'Hala California Acad of Sciences malaise trap in high altitude rainforest, MA-02-09A-06
CASENT 2004753; PSUC_FEM 79715	CAS	male	MADAGASCAR: Procince d'Antananarivo 3 km 41ºNE Andranomay, 11.5km 147º SSE Anjozorobe elev 1300m, 5-13.Dec. 2000 18º28'24"S 47º57'36"E coll. Fisher,

			Griswold et al. California Acad. of Sciences montane rainforest, Malaise trap, coll. code: BLF2375
CASENT 2022986; PSUC_FEM 79712	CAS	male	MADAGASCAR: Province Fianarantsoa, Ranomafana JIRAMA water works 21°14.91'S, 47°27.13'E 21-24 December 2001 collector: R. Harin'Hala California Acad of Sciences Malaise trap near river elev 690m MA-02-09D-08
CASENT 2044895; PSUC_FEM 79709	CAS	male	Madagascar: Province Fianarantsoa, Parc National Ranomafana, radio tower at forest edge. elev. 1030 24 May -- 4 June 2002 21°15.05" S, 47° 24.43' E collector: R. Harin'Hala California Acad. of Sciences Malaise, mixed tropical forest MA-02-09B-30
CASENT 2041945; PSUC_FEM 79717	CAS	male	Madagascar: Province Fianarantsoa, Parc National Ranomafana, Vohiparara at broken bridge, el 1110m 6-15 December 2001 21º13.57'S 47º22.19'E collector R.Harin'Hala California Acad of Sciences malaise trap in high altitude rainforest, MA-02-09A-06
CASENT 2053573	CAS	female	MADAGASCAR: Fianarantsoa Parc National de Ranomafana Vatoharanana River 4.1 km 231º SW Ranomafana elev. 1100m 27-31Mar 2003 21º 17'24"S 047º26'00" E California Academy of Sciencescoll. Fisher, Griswold et. al. malaise trap, montane rainforestcode: BLF8397
CASENT 2004749	CAS	female	MADAGASCAR: Procince d'Antananarivo 3 km 41ºNE Andranomay, 11.5km 147º SSE Anjozorobe elev 1300m, 5-13.Dec. 2000 18º28'24"S 47º57'36"E coll. Fisher, Griswold et al. California Acad. of Sciences montane rainforest, Malaise trap, coll. code: BLF2375
CASENT 2002180	CAS	female	MADAGASCAR: Procince d'Antananarivo 3 km 41ºNE Andranomay, 11.5km 147º SSE Anjozorobe elev 1300m, 5-13.Dec. 2000 18º28'24"S 47º57'36"E coll. Fisher, Griswold et al. California Acad. of Sciences montane rainforest, Malaise trap, coll. code: BLF2375
CASENT 2053365	CAS	female	MADAGASCAR: Province Fianarantsoa, Parc National Ranomafana, radio tower at forest edge, elev 1130m. 7-17 July 2003. 21°15.05'S, 47°24.43'E; ; collector: R. Harin'Hala California Academy of Sciences, malaise, mixed tropical forest MA-02-09B-66
CASENT 2053574	CAS	female	MADAGASCAR: Fianarantsoa Parc National de Ranomafana Vatoharanana River 4.1 km 231º SW Ranomafana elev. 1100m 27-31Mar 2003 21º 17'24"S

			047º26'00" E California Academy of Sciencescoll. Fisher, Griswold et. al. malaise trap, montane rainforestcode: BLF8397
CASENT 2009143	CAS	female	MADAGASCAR: Province Diego-Suarez, Parc National Montagne d'Ambre 1125m 29 Jan to 11 Feb 2001 12°31'13"S, 49°10'45"E R. Harin'Hala collector malaise trap MA-01-01D-03
CASENT 2040890	CAS	female	Madagascar: Province d'Antananarivo 3 km 41ºNE Andranomay, 11.5 km 147º SSE Anjozorobe elev 1300m, 5-13 Dec 2000 18º28'24"S 47º57'36"E coll. Fisher, Griswold et al California Acad. of Sciences montane rainforest, malaise trap, coll. code: BLF2372
CASENT 2040898	CAS	female	Madagascar: Province d'Antananarivo 3 km 41ºNE Andranomay, 11.5 km 147º SSE Anjozorobe elev 1300m, 5-13 Dec 2000 18º28'24"S 47º57'36"E coll. Fisher, Griswold et al California Acad. of Sciences montane rainforest, malaise trap, coll. code: BLF2372
CASENT 2044995	CAS	female	Madagascar, Province Fianarantsoa, Parc National Ranomafana, radio tower at forest edge. elev. 1130 m 15-27 April 2003 21°15.05'S, 47°24.43' E Collector: R. Harin'Hala California Academy of Sciences. Malaise, mixed tropical forest. MA-02-09B-58
CASENT 2000886	CAS	female	MADAGASCAR: Province d'Antanenarivo, Reserve Speciale d'Ambohitantely. Foret d Ambohitantely. 20.9 km. 72°NE d'Ankazobe el 1410m. 17-22 April 2001. 18°13'31" S 47°17'13" E coll. Fisher, Griswold et al. California Academy of Sciences. malaise trap - in montane rainforest. code: BLF3693
CASENT 2009144	CAS	female	MADAGASCAR: Province Diego-Suarez, Parc National Montagne d'Ambre 1125m 29 Jan to 11 Feb 2001 12°31'13"S, 49°10'45"E R. Harin'Hala collector malaise trap MA-01-01D-03
CASENT 2044509	CAS	female	Madagascar: Toamasina Province, botanic garden near entrance to Andasibe National Park, 8-16 October 2001 18º55.58'S, 48º24,47'E collector: R. Harin'Hala California Acad. of Sciences Malaise trap- tropical forest elev 1025m, MA-01-08B-15
CASENT 2004747	CAS	female	MADAGASCAR: Procince d'Antananarivo 3 km 41ºNE Andranomay, 11.5km 147º SSE Anjozorobe elev 1300m, 5-13.Dec. 2000 18º28'24"S 47º57'36"E coll. Fisher, Griswold et al. California Acad. of Sciences montane

			rainforest, Malaise trap, coll. code: BLF2375
CASENT 2044824	CAS	male	MADAGASCAR: Province Fianarantsoa, Parc National Ranomafana, radio tower at forest edge; -21.251,47.407; 1130m; 12-19.iii.2002; MT; MA-02-09B-20; R. Harin'Hala
CASENT 2040905	CAS	male	Madagascar: Province d'Antananarivo 3 km 41ºNE Andranomay, 11.5 km 147º SSE Anjozorobe elev 1300m, 5-13 Dec 2000 18º28'24"S 47º57'36"E coll. Fisher, Griswold et al California Acad. of Sciences montane rainforest, malaise trap, coll. code: BLF2372
CASENT 2040889	CAS	female	Madagascar: Province d'Antananarivo 3 km 41ºNE Andranomay, 11.5 km 147º SSE Anjozorobe elev 1300m, 5-13 Dec 2000 18º28'24"S 47º57'36"E coll. Fisher, Griswold et al California Acad. of Sciences montane rainforest, malaise trap, coll. code: BLF2372
CASENT 2053393	CAS	male	Madagascar: Toliara Prov., Fiherenana, el. 100m. 23°10'37"S 043°57'39"E 22-28 Oct. 2002. California Academy of Sciences. coll: Frontier Wilderness Project, malaise trap in gallery forest. MGF041

Conostigmus missyhazenae (Mdsp. 7)

Identifier(s)	Repository	Sex	Collecting event
PSUC_FEM 000079747; CASENT 2046183	MRAC	male	Madagascar: Prov. Fianarantsoa Parc National Ranomafana: Belle Vue at Talatakely; 1020m; -21.2655, 47.4202; 22-28.xi.2001; Malaise trap in secondary tropical forest. Collector: R. Hanin'Hala. MA-02-09C-04
PSUC_FEM 000079733; CASENT 2046019	CAS	male	Madagascar: Province Fianarantsoa, Parc National Ranomafana, Belle Vue at Talatakely, elev 1020m 12-19 February 2002 21°15.99'S, 47°25.21'E collector: R. Harin'Hala California Acad of Sciences Malaise, secondary tropical forest MA-02-09C-16
PSUC_FEM 000079731; CASENT 2046021	CAS	male	Madagascar: Province Fianarantsoa, Parc National Ranomafana, Belle Vue at Talatakely, elev 1020m 12-19 February 2002 21°15.99'S, 47°25.21'E collector: R. Harin'Hala California Acad of Sciences Malaise, secondary tropical forest MA-02-09C-16
CASENT 2004752	CAS	female	MADAGASCAR: Procince d'Antananarivo 3 km 41ºNE Andranomay, 11.5km 147º SSE Anjozorobe elev 1300m, 5-13.Dec. 2000 18º28'24"S 47º57'36"E coll. Fisher, Griswold et al. California Acad. of Sciences montane

			rainforest, Malaise trap, coll. code: BLF2375
CASENT 2002183	CAS	female	MADAGASCAR: Procince d'Antananarivo 3 km 41ºNE Andranomay, 11.5km 147º SSE Anjozorobe elev 1300m, 5-13.Dec. 2000 18º28'24"S 47º57'36"E coll. Fisher, Griswold et al. California Acad. of Sciences montane rainforest, Malaise trap, coll. code: BLF2375

Conostigmus pseudobabaiax (Mad sp. 11)

Identifier(s)	Repository	Sex	Collecting event
CASENT CASENT_2040937; PSUC_FEM 79706	CAS	male	MADAGASCAR: Toliara Prov: Res. Speciale d'Ambohijanahary: Foret d' Ankazotsihitafototra: 35.2km; NW Ambaravaranala; 1050m; 18º16'00"S, 45º24'24"E; 13-17.i.2003; MT; MISC BLF7020; Fisher, Griswold, et al. Sifted litter-in mountain rainforest
PSUC_FEM 000079744; CASENT 2053690	CAS	male	MADAGASCAR: Province Fianarantsoa,Ranomafana JIRAMA water works 21°14.91'S, 47°27.13'E 16 Oct-8 Nov 2001 collector: R. Harin'Hala California Acad of Sciences malaise trap near river elev 690 m MA-02-09D-01
PSUC_FEM 000079736; CASENT 2045087	CAS	male	MADAGASCAR: Province Fianarantsoa,Parc National Ranomafana, radio tower at forest edge, 1130m 14-21 January 2002 21°15.05'S, 47°24.43'E collector: R. Harin'Hala California Acad of Sciences malaise, mixed tropical forest MA-02-09B-12
CASENT 2053425	CAS	male	MADAGASCAR: Province Fianarantsoa, Parc National Ranomafana, Belle Vue at Talatakely elev 1020m 10-21 March 2003 21°15.99'S, 47°25.21'E collector: R. Harin'Hala California Acad of Sciences Malaise, secondary tropical forest MA-02-09C-56
CASENT 2046151	CAS	male	Madagascar, Province Fianarantsoa, Parc National Ranomafana, Belle Vue at Talatakely elev. 1120 m 6-15 December 2001 21°15.99’S, 47°25.21’ E Collector: R. Harin'Hala California Academy of Sciences. Malaise, secondary tropical forest. MA-02-09C-06
CASENT 2053381	MRAC	female	MADAGASCAR: Toamasina Montagne d'Anjanaharibe 18.0 km 21° NNE Ambinanitelo elev 470m 8-12 Mar 2003 15°11'18"S, 049°36'54"E California Acad. of Sciences coll. Fisher, Griswold et al yellow pan trap, in rainforest collection code: BLF8004
CASENT 2041943	CAS	female	Madagascar: Province Fianarantsoa, Parc National

			Ranomafana, Vohiparara at broken bridge, el 1110m 6-15 December 2001 21º13.57'S 47º22.19'E collector R.Harin'Hala California Acad of Sciences malaise trap in high altitude rainforest, MA-02-09A-06
CASENT 2032774	MRAC	male	Madagascar: Province Fianarantsoa, Parc National Ranomafana, Belle Vue at Talatakely. Elev. 1020m. 24 July -- 4 August 2002. 21°15.99' S, 47° 25.21' E Collector: R. Harin'Hala California Academy of Sciences Malaise, secondary tropical forest. MA-02-09C-36
CASENT 2046097	CAS	female	Madagascar:Province Fianarantsoa, Parc National Ranarantsoa, Belle Vue at Talatakely. elev. 1020m 10-14 January 2002. 21°15.99' S, 47°25.21' E Collector: R. Harin'Hala California Academy of sciences. Malaise, secondary tropical forest. MA-02-09C-11
CASENT 2006450	CAS	female	MADAGASCAR: Fianarantsoa Province Ranomafana National Park Talatakely area. flight trap mixed tropical forest. 900m. 21.25041 S 47.41945 E. 9-19 January 2001. D. H. & K. M. Kavanaugh, R. L.. Brett, E. Elsom and F. Vargas. Calif. Academy Sciences.
CASENT 2006451	CAS	female	MADAGASCAR: Fianarantsoa Province Ranomafana National Park Talatakely area. flight trap mixed tropical forest. 900m. 21.25041 S 47.41945 E. 9-19 January 2001. D. H. & K. M. Kavanaugh, R. L.. Brett, E. Elsom and F. Vargas. Calif. Academy Sciences.
CASENT 2053382	CAS	female	MADAGASCAR: Toamasina Montagne d'Anjanaharibe 18.0 km 21° NNE Ambinanitelo elev 470m 8-12 Mar 2003 15°11'18"S, 049°36'54"E California Acad. of Sciences coll. Fisher, Griswold et al yellow pan trap, in rainforest collection code: BLF8004

Conostigmus toliaraensis (Mad sp. 10)

**Identifier(s)**	**Repository**	**Sex**	**Collecting event**
CASENT 2053309	CAS	male	MADAGASCAR: Toliara Prov: Res. Speciale d'Ambohijanahary: Foret d' Ankazotsihitafototra: 35.2km; NW Ambaravaranala; 1050m; 18º16'00"S, 45º24'24"E; 13-17.i.2003; MT; MISC BLF7019; Fisher, Griswold, et al. California Academy of Sciences. Yellow pan trap- in montane rainforest.
CASENT 2053311	CAS	female	MADAGASCAR: Toliara Prov: Res. Speciale d'Ambohijanahary: Foret d' Ankazotsihitafototra: 35.2km; NW Ambaravaranala; 1050m; 18º16'00"S, 45º24'24"E;

			13-17.i.2003; MT; MISC BLF7019; Fisher, Griswold, et al. California Academy of Sciences. Yellow pan trap- in montane rainforest.
CASENT 2053310	CAS	female	MADAGASCAR: Toliara Prov: Res. Speciale d'Ambohijanahary: Foret d' Ankazotsihitafototra: 35.2km; NW Ambaravaranala; 1050m; 18º16'00"S, 45º24'24"E; 13-17.i.2003; MT; MISC BLF7019; Fisher, Griswold, et al. California Academy of Sciences. Yellow pan trap- in montane rainforest.
CASENT 2053452	CAS	female	MADAGASCAR: Toliara Province. Foret Classee d'Analavelona, 29.2 km 343 NNW Mahaboboka elev. 1100m 18-22 Feb 2003 22º 40' 30" S 044º 11' 24" E coll. Fisher, Griswold et. al. California Academy of Sciences. Malaise trap, in montane rainforest. Code: BLF7818
CASENT 2041206	CAS	female	MADAGASCAR: Toliara Province. Foret Classee d'Analavelona, 29.2 km 343 NNW Mahaboboka elev. 1100m 18-22 Feb 2003 22º40' 30" S 044º 11' 24" E coll. Fisher, Griswold et. al. California Academy of Sciences. sifted litter, in montane rainforest. Code: BLF7820
CASENT 2040983	CAS	female	MADGASCAR: Toliara Prov. Res. Speciale d' Ambohijanahary, Foret d'Ankazotsihtafototra, 34.6 km 314º NW Ambaravaranala elev. 1100m 16 Jan 2003 18º15'36"S, 045º25'06"E; coll. Fisher, Griswold, et al. California Academy of Sciences. sifted litter-leaf mold/rotten wood montane rainforest BLF7086
CASENT 2040934	CAS	female	MADAGASCAR: Toliara Prov: Res. Speciale d'Ambohijanahary: Foret d' Ankazotsihitafototra: 35.2km; NW Ambaravaranala; 1050m; 18º16'00"S, 45º24'24"E; 13-17.i.2003; MT; MISC BLF7018; Fisher, Griswold, et al. California Academy of Sciences. Pitfall trap- in montane rainforest.
CASENT 2040935	CAS	female	MADAGASCAR: Toliara Prov: Res. Speciale d'Ambohijanahary: Foret d' Ankazotsihitafototra: 35.2km; NW Ambaravaranala; 1050m; 18º16'00"S, 45º24'24"E; 13-17.i.2003; MT; MISC BLF7018; Fisher, Griswold, et al. California Academy of Sciences. Pitfall trap- in montane rainforest.
CASENT 2040936	CAS	female	MADAGASCAR: Toliara Prov: Res. Speciale d'Ambohijanahary: Foret d' Ankazotsihitafototra: 35.2km; NW Ambaravaranala; 1050m; 18º16'00"S, 45º24'24"E; 13-17.i.2003; MT; MISC BLF7018; Fisher, Griswold, et

			al. California Academy of Sciences. Pitfall trap- in montane rainforest.
CASENT 2009754	CAS	female	Madagascar:Province d' Antsiranana, botanic garden near the entrance to Andasibe National Park. 1-5 Sept. 2001.18°55.58' S. 48°24.47' E. coll. R. Harin'Hala. California Academy of Sciences. malaise trap- tropical forest. elev. 1025m. MA-01-08B-11
