# Supplementary material for: Malagasy Conostigmus (Hymenoptera: Ceraphronoidea) and the secret of scutes
Source: PeerJ. 2016 Dec 13;4:e2682. doi: 10.7717/peerj.2682 (PMC5157207; doi:10.7717/peerj.2682)
Supplement: Supplemental Information 7 [file peerj-04-2682-s007.pdf]

Table S3: URI table (Seltmann et al. 2013) containing anatomical terms, definitions and uniform resource identifiers of Hymenoptera specific classes in the Hymenoptera Anatomy Ontology and abbreviations (http://hymao.org).

Abbreviation	Term	Concept	URI
	Metanoto-propodeo-metapecto-mesop	The sclerite that is connected anterolaterally and anteroventrally with the pronotum and anterodorsally with the mesoscutellum and the mesopo	http://purl.obolibrary.org/obo/HAO_0001921
	S9	The sternite that is connected posteriorly to the cupula via muscles.	http://purl.obolibrary.org/obo/HAO_0002053
	abdominal tergum	The tergum that is located in the abdomen.	http://purl.obolibrary.org/obo/HAO_0001426
	anatomical line	Non-material anatomical entity of one dimension, which forms a boundary of an anatomical surface or is a modulation of an anatomical surface	http://purl.obolibrary.org/obo/HAO_0000008
	antecostal sulcus	The sulcus that corresponds to the antecosta.	http://purl.obolibrary.org/obo/HAO_0000099
	antecostal sulcus of the first abdomina	The sulcus that corresponds to the metaphragma.	http://purl.obolibrary.org/obo/HAO_0001853
	antenna	The appendage that is composed of ringlike sclerites and the anatomical structures encircled by these sclerites and that is articulated with the cr	http://purl.obolibrary.org/obo/HAO_0000101
	antennal rim	The rim that surrounds the antennal foramen.	http://purl.obolibrary.org/obo/HAO_0000103
asr	antennal scrobe	The scrobe that is located dorsally of the antennal foramen and is for the reception of the antenna.	http://purl.obolibrary.org/obo/HAO_0001432
	anterior discrimenal pit	The pit at the anteriormost point of the mesodiscrimen.	http://purl.obolibrary.org/obo/HAO_0002393
	anterior mesopleuro-mesofurcal musc	The mesofurcal muscle that originates from the mesopleuron and inserts distally on the mesofurca.	http://purl.obolibrary.org/obo/HAO_0000118
app	anteromedian projection of the metan	The median projection on the anterior region of the metanoto-propodeo-metapecto-mesopectal complex.	http://purl.obolibrary.org/obo/HAO_0002397
aps	apical parossiculal seta	The seta that is located close to the distoventral margin of the gonostyle/volsella complex just laterally of the gonostyle/volsella complex-gonos	http://purl.obolibrary.org/obo/HAO_0000138
	apodeme	The process that is internal.	http://purl.obolibrary.org/obo/HAO_0000142
	area	The anatomical structure of the cuticle that is delimited by material or immaterial anatomical entities.	http://purl.obolibrary.org/obo/HAO_0000146
	articulation	The anatomical cluster that is composed of two adjacent articular surfaces.	http://purl.obolibrary.org/obo/HAO_0000151
axc	axillular carina	The axillular line that is a carina.	http://purl.obolibrary.org/obo/HAO_0000161
	body	The anatomical cluster that is composed of the whole organism but which excludes the antennae, legs and wings.	http://purl.obolibrary.org/obo/HAO_0000182
	body length	The anatomical line between the anteriormost pont of the cranium and the posteriormost point of the metasoma.	http://purl.obolibrary.org/obo/HAO_0002413
	carina	The process that is elongate and external.	http://purl.obolibrary.org/obo/HAO_0000188
	clypeal carina	The carina that is longitudinal and is located on the clypeus.	http://purl.obolibrary.org/obo/HAO_0001187
	clypeus	The area that corresponds to the site of origin of the clypeo-epipharyngeal muscle.	http://purl.obolibrary.org/obo/HAO_0000212
	conjunctiva	The area of the cuticle that is weakly sclerotized, with thin exocuticle.	http://purl.obolibrary.org/obo/HAO_0000221
	corner	The projection that is located at the intersection of two or more edges.	http://purl.obolibrary.org/obo/HAO_0000223
	coxa	The leg segment that is connected to the body and to the trochanter via conjunctivae and muscles.	http://purl.obolibrary.org/obo/HAO_0000228
	cranium	The sclerite that is articulated with the cervical prominence, the scapes and the mandibles.	http://purl.obolibrary.org/obo/HAO_0000234
cup	cupula	The sclerite that is connected via conjunctiva and attached via muscles to abdominal tergum 9 and the gonostyle/volsella complex.	http://purl.obolibrary.org/obo/HAO_0000238
	cuspis	The projection that is located apicolaterally on the parossiculus and is adjacent to the digitus.	http://purl.obolibrary.org/obo/HAO_0000239
	depression	The area that is external, concave, point-like and does not correspond to an apodeme.	http://purl.obolibrary.org/obo/HAO_0000241
	discrimenal lamella	The ridge that is median, longitudinal and arises anteriorly from the base of the furca.	http://purl.obolibrary.org/obo/HAO_0000245
	distal projection of the parossiculus	The distal projection of the volsella that corresponds to the apical parossiculal seta.	http://purl.obolibrary.org/obo/HAO_0002386
	distal projection of the penisvalva	The projection that is at the distal end of the penisvalva.	http://purl.obolibrary.org/obo/HAO_0002391
	dorsal apodeme of penisvalva	The apodeme that is located proximodorsally on the aedeagus and is the site of insertion of the distodorsal gonostyle/volsella complex-penisval	http://purl.obolibrary.org/obo/HAO_0002036
dc	dorsomedian conjunctiva of the gonos	The conjunctiva that is linear and extends medially on the dorsal region of the gonostyle/volsella complex.	http://purl.obolibrary.org/obo/HAO_0002040
	edge	The margin that extends along the border of two areas that are oriented differently.	http://purl.obolibrary.org/obo/HAO_0000285
	epicnemial carina	The carina that delimits posteriorly the epicnemium.	http://purl.obolibrary.org/obo/HAO_0000292
	epicnemium	The scrobe that is located anteroventrally on the mesopectus and accommodates the procoxa.	http://purl.obolibrary.org/obo/HAO_0000294
	eye	The compound organ that is composed of ommatidia.	http://purl.obolibrary.org/obo/HAO_0000217
	eye margin	The margin of the compound eye.	http://purl.obolibrary.org/obo/HAO_0000672
	facial apodeme	The apodeme that is dorsal to the intertorular line and ventral to the anterior ocellus.	http://purl.obolibrary.org/obo/HAO_0002387
	facial pit	The pit that overlaps with the facial apodeme.	http://purl.obolibrary.org/obo/HAO_0000319
	facial process	The process that is located on the upper face dorsal to the intertorular carina and corresponds internally to a conical apodeme and not the tentori	http://purl.obolibrary.org/obo/HAO_0002384

	first flagellomere	The flagellomere that is proximally attached to the pedicel.	http://purl.obolibrary.org/obo/HAO_0001148
	flagellomere	The annulus that is located distally of the pedicel.	http://purl.obolibrary.org/obo/HAO_0000342
	flagellum	The anatomical cluster composed of flagellomeres.	http://purl.obolibrary.org/obo/HAO_0000343
	flange	The projection that is lamella-like and is located on a rim, carina, apodeme or edge.	http://purl.obolibrary.org/obo/HAO_0000344
	fore leg	The leg that is located on the prothorax.	http://purl.obolibrary.org/obo/HAO_0000349
	fourth flagellomere	The flagellomere that is located immediately distal to the third flagellomere.	http://purl.obolibrary.org/obo/HAO_0001896
	frons	The area that is between the epistomal line and the anterior ocellus and limited laterally by the inner margin of compound eye.	http://purl.obolibrary.org/obo/HAO_0001523
	gonoforceps	The sclerite that is located distally of the cupula and dorsolaterally of the volsella, surrounds the aedeagus dorsolaterally and does not connecte	http://purl.obolibrary.org/obo/HAO_0000381
	gonossiculal muscle	The male genitalia muscle that inserts on the gonossiculus.	http://purl.obolibrary.org/obo/HAO_0002045
gs	gonostipes	The sclerite that is located dorsolaterally on the gonostyle/volsella complex, is connected to the distal margin of the cupula, to the proximal mar	http://purl.obolibrary.org/obo/HAO_0000386
gvs	gonostyle-volsella complex	The anatomical cluster that is composed of the sclerites that are located distally of the cupula and surround the aedeagus.	http://purl.obolibrary.org/obo/HAO_0002033
hrp	harpe	The sclerite that is located distally on the gonostyle/volsella complex and does not connected to the cupula and to the volsella by conjunctiva or	http://purl.obolibrary.org/obo/HAO_0000395
	harpe length	The anatomical line between the distalmost point of the gonostyle volsella complex and the distalmost point of the harpe in lateral view.	http://purl.obolibrary.org/obo/HAO_0002409
	harpe width	The width of the harpe in lateral view.	http://purl.obolibrary.org/obo/HAO_0002406
	head	The tagma that is located anterior to the thorax.	http://purl.obolibrary.org/obo/HAO_0000397
	head width	The anatomical line that is the longest horizontal diameter of the cranium in frontal view.	http://purl.obolibrary.org/obo/HAO_0002268
	hind leg	The leg that is located on the metathorax.	http://purl.obolibrary.org/obo/HAO_0000399
	hyperoccipital carina	The carina that extends on the vertex between the outer orbits.	http://purl.obolibrary.org/obo/HAO_0000406
	inflection	The anatomical cluster that is composed of a ridge that extends along the margin of a sclerite and the marginal area of the sclerite delimited fro	http://purl.obolibrary.org/obo/HAO_0000418
	interorbital space	The interocular distance that is the shortest between the inner margin of compound eyes.	http://purl.obolibrary.org/obo/HAO_0000432
ita	intertorular area	The median area of the face between the dorsal (posterior) margin of the clypeus and the intertolural carina.	http://purl.obolibrary.org/obo/HAO_0002383
	intertorular carina	The carina that extends between the dorsal margin of the antennal rims.	http://purl.obolibrary.org/obo/HAO_0000439
	labial palp	The anatomical structure that is distal to the proximal margin of the first sclerite of the labial palp.	http://purl.obolibrary.org/obo/HAO_0000450
	lateral ocellar line	The anatomical line that is the shortest between the margins of the median and lateral ocelli.	http://purl.obolibrary.org/obo/HAO_0000480
	lateral ocellus	The ocellus that is paired.	http://purl.obolibrary.org/obo/HAO_0000481
	lateral propodeal carina	The carina that is oblique and arises submedially from the anterior margin of the metapectal-propodeal complex and extends to the posterior pr	http://purl.obolibrary.org/obo/HAO_0000486
	leg	The appendage that is composed of the coxa and all distal leg segments and is connected to the pectus.	http://purl.obolibrary.org/obo/HAO_0000494
	line	The anatomical structure that is linear.	http://purl.obolibrary.org/obo/HAO_0001586
	mandible	The appendage that is encircled by one sclerite that is connected to the cranium proximolaterally and to the maxillo-labial complex proximome	http://purl.obolibrary.org/obo/HAO_0000506
	margin	The line that delimits the periphery of an area.	http://purl.obolibrary.org/obo/HAO_0000510
	maxillary palp	The anatomical structure that is distal to the proximal margin of the first sclerite of the maxillary palp.	http://purl.obolibrary.org/obo/HAO_0000515
	median facial keel	The carina whose dorsal (posterior) region overlaps the facial pit.	http://purl.obolibrary.org/obo/HAO_0002388
mfc	median flange of occipital carina	The flange that is median and on the occipital carina.	http://purl.obolibrary.org/obo/HAO_0002389
mml	median mesoscutal sulcus	The sulcus that is a median mesoscutal line and that is adjacent to the (internal) median mesoscutal ridge.	http://purl.obolibrary.org/obo/HAO_0000523
	median gonostyle/volsella complex-v	The gonostyle/volsella complex-volsellal muscle that arises medially of the submedian conjunctiva on the distoventral margin of gonostyle/vol	http://purl.obolibrary.org/obo/HAO_0000473
	mesofurcal muscle	The thoracic muscle that inserts on the mesofurca.	http://purl.obolibrary.org/obo/HAO_0001787
	mesometapleural sulcus	The sulcus that crosses the mesometapleuron from the posterolateral margin of the mesocoxal foramen to the mesopleural pit.	http://purl.obolibrary.org/obo/HAO_0000553
	mesopectus	The sclerite that is U-shaped in cross section, connected anteriorly with the pronotum and the propectus, dorsally with the basalare, the mesono	http://purl.obolibrary.org/obo/HAO_0000557
	mesopleuron	The lateral (vertical) area that is anterior to the mesometapleural sulcus and posterior to the pronotum.	http://purl.obolibrary.org/obo/HAO_0002363
	mesopleuron length	The anatomical line that extends between the lateral end of the epicnemial carina and the postpectal carina and is parallel to the lateroventral m	http://purl.obolibrary.org/obo/HAO_0002403
	mesoscutellar-axillar complex	The area that is located posteriorly of the transscutal line and is composed of the axillae and the mesoscutellum.	http://purl.obolibrary.org/obo/HAO_0000572
	mesoscutellum	The scutellum that is located on the mesonotum.	http://purl.obolibrary.org/obo/HAO_0000574
	mesoscutum	The area that is located anterior to the transscutal articulation.	http://purl.obolibrary.org/obo/HAO_0001490
	mesosoma	The anatomical cluster that is composed of the prothorax, mesothorax and the metapectal-propodeal complex.	http://purl.obolibrary.org/obo/HAO_0000576
	mesotibia	The tibia that is located on the mid leg.	http://purl.obolibrary.org/obo/HAO_0001351
	metacoxa	The coxa that is located on the hind leg.	http://purl.obolibrary.org/obo/HAO_0000587

	metafemur	The femur that is located on the hind leg.	http://purl.obolibrary.org/obo/HAO_0001140
	metanotum	The alinotum that is located in the metathorax, is connected with the mesoscutellum and the mesopostnotum anteriorly and the acrotergite of th	http://purl.obolibrary.org/obo/HAO_0000603
	metapleural carina	The carina that delimits the metapleuron dorsally from the propodeum, extends from just ventral of the metapleural arm to the metacoxal articu	http://purl.obolibrary.org/obo/HAO_0000609
	metapleural pit	The pleural pit that is corresponds to the metapleural apodeme.	http://purl.obolibrary.org/obo/HAO_0000612
	metapleuron	The lateral (vertical) area that is posterior to the mesometapleural sulcus and anterior to the metapleural carina.	http://purl.obolibrary.org/obo/HAO_0002360
	metasoma	The tagma that is connected anteriorly to the metapectal-propodeal complex at the propodeal foramen and consists of abdominal segments.	http://purl.obolibrary.org/obo/HAO_0000626
	metathorax	The thoracic segment that is located between the mesothorax and the first abdominal tergum and is delimited by the metanotum and the metape	http://purl.obolibrary.org/obo/HAO_0000630
	mouthparts	The anatomical cluster that is composed of the labrum, epipharyngeal wall, hypopharyngeal wall (including the sitophore), mandibles, maxillae	http://purl.obolibrary.org/obo/HAO_0000639
	muscle	The portion of tissue that is composed of contractile fibers.	http://purl.obolibrary.org/obo/HAO_0000641
	ninth flagellomere	The flagellomere that is located immediately distal to the eighth flagellomere.	http://purl.obolibrary.org/obo/HAO_0001900
	notaulus	The line that extends submedially along the mesoscutum and corresponds to the median border of the site of origin of the first mesopleuro-mes	http://purl.obolibrary.org/obo/HAO_0000647
	notch	The part of the margin of a sclerite that is concave.	http://purl.obolibrary.org/obo/HAO_0000648
	occipital carina	The carina that surrounds dorsolaterally the occiput.	http://purl.obolibrary.org/obo/HAO_0000653
	ocellar triangle	The area that is limited posteriorly by the posterior ocellar line and laterally by the lateral ocellar line.	http://purl.obolibrary.org/obo/HAO_0000430
	ocellus	The multi-tissue structure that is located on the top of the head, composed of the corneal lens, pigment cell, rhabdoms and synaptic plexus.	http://purl.obolibrary.org/obo/HAO_0000661
	ocular ocellar line	The anatomical line that is shortest and connects the compound eye and the lateral ocellus.	http://purl.obolibrary.org/obo/HAO_0000662
	palp	The anatomical cluster that is composed of the palpal segments.	http://purl.obolibrary.org/obo/HAO_0000683
pss	parossiculus	The sclerite that is connected via conjunctiva distomedially to the gonostipes, and articulates with the gonossiculus.	http://purl.obolibrary.org/obo/HAO_0000703
	patch	The area that is round and differs from surrounding regions in sculpture, setae, and/or pigmentation.	http://purl.obolibrary.org/obo/HAO_0000704
	pedicel	The antennal segment that is connected proximally to the scape and distally to the flagellum.	http://purl.obolibrary.org/obo/HAO_0000706
	penisvalva	The sclerite that is in the middle of the external male genitalia, surrounds the distal part of the ductus ejaculatorius and the endophallus.	http://purl.obolibrary.org/obo/HAO_0000707
	penisvalval muscle	The male genitalia muscle that inserts on the penisvalva.	http://purl.obolibrary.org/obo/HAO_0002051
	penisvalvo-gonossiculal muscle	The gonossiculal muscle that arises from the penisvalva.	http://purl.obolibrary.org/obo/HAO_0000709
	petiole neck	The anterior area of the petiole that is narrower than the posterior region in dorsal view.	http://purl.obolibrary.org/obo/HAO_0002411
	pharynx	The anatomical space that is located proximal to the cibarium.	http://purl.obolibrary.org/obo/HAO_0001740
	pleural pit	The pit that is located on the pleuron and corresponds to the pleural apophysis.	http://purl.obolibrary.org/obo/HAO_0000723
	pleural pit line	The anatomical line between the mesopleural and metapleural pits.	http://purl.obolibrary.org/obo/HAO_0002374
	posterior ocellar line	The anatomical line that is the shortest between the margin of the lateral ocelli.	http://purl.obolibrary.org/obo/HAO_0000759
	postoccipital carina	The rim that surrounds the occipital foramen.	http://purl.obolibrary.org/obo/HAO_0001810
pcc	postocellar carina	The carina that connects the posterior margins of the left and right lateral ocellar fovea.	http://purl.obolibrary.org/obo/HAO_0002385
poc	preoccipital carina	The carina that is transverse and extends posteriorly of the lateral ocelli and compound eye and delimits anteriorly the preoccipital lunula.	http://purl.obolibrary.org/obo/HAO_0000805
pos	preoccipital furrow	The sulcus that arises medially from the anterior margin of the occipital carina and extends towards the anterior ocellus.	http://purl.obolibrary.org/obo/HAO_0000806
	preoccipital lunula	The area that is concave, is located on the vertex and is limited anteriorly by the preoccipital carina and posteriorly by the occipital carina.	http://purl.obolibrary.org/obo/HAO_0000807
	process	The area on the sclerite that is raised.	http://purl.obolibrary.org/obo/HAO_0000822
	projection	The process that is located on an edge.	http://purl.obolibrary.org/obo/HAO_0000829
	pronotum	The notum that is located in the prothorax.	http://purl.obolibrary.org/obo/HAO_0000853
	propodeal carina	The carina that is located on the propodeum.	http://purl.obolibrary.org/obo/HAO_0000864
	propodeum	The area of the metapectal-propodeal complex that is located posterior to the metapleural carina.	http://purl.obolibrary.org/obo/HAO_0001249
	The anatomical line that connects the	The area that is located dorsomedially on the metapectal-propodeal complex.	http://purl.obolibrary.org/obo/HAO_0001994
	protibia	The tibia that is located on the fore leg.	http://purl.obolibrary.org/obo/HAO_0000350
gs-hrp	proximal gonostyle/volsella co	The gonostyle/volsella complex-harpal muscle that inserts on the median wall of the harpe and arises proximally of the	http://purl.obolibrary.org/obo/HAO_0000926
pdc	proximodorsal notch of cupula	The notch that is medially on the proximodorsal margin of the cupula and is delimited laterally by the site of origins of the dorsomedial cupulo-	http://purl.obolibrary.org/obo/HAO_0002048
ppc	proximolateral projection of the cupul	The projection that is between the proximodorsal notch of cupula and the proximoventral notch of the cupula.	http://purl.obolibrary.org/obo/HAO_0002394
	region	The anatomical structure that is delimited by at least one immaterial anatomical entity.	http://purl.obolibrary.org/obo/HAO_0000893
	ridge	The apodeme that is elongate.	http://purl.obolibrary.org/obo/HAO_0000899
	rim	The carina that extends along the margin or edge of a sclerite.	http://purl.obolibrary.org/obo/HAO_0000900

	row	The anatomical cluster that is composed of repeated units of anatomical structures.	http://purl.obolibrary.org/obo/HAO_0000901
	row of setae	The row that is composed of setae.	http://purl.obolibrary.org/obo/HAO_0000903
	scape	The antennal segment that is proximal to the pedicel and is connected to the head via the radicle.	http://purl.obolibrary.org/obo/HAO_0000908
	scrobe	The area that is impressed and is for the reception or concealment of another sclerite.	http://purl.obolibrary.org/obo/HAO_0000912
	sculpture	The area that is located on the sclerite and that is composed of repetitive anatomical structures.	http://purl.obolibrary.org/obo/HAO_0000913
	scutoscutellar sulcus	The sulcus that extends along the scutoscutellar suture.	http://purl.obolibrary.org/obo/HAO_0000919
	sensillar patch	The patch that differs from surrounding region by presence of sensilla.	http://purl.obolibrary.org/obo/HAO_0000931
	sensillar patch of the male flagellomer	The sensillar patch that is located on the ventral surface of the male flagellomere.	http://purl.obolibrary.org/obo/HAO_0000932
	sensillar ring of harpe	The row of setae on the distomedial harpal wall that is parallel to the harpal margin.	http://purl.obolibrary.org/obo/HAO_0002392
	seta	The sensillum with a hair-like cuticular component.	http://purl.obolibrary.org/obo/HAO_0002299
	setal pit	The impression with a centered seta.	http://purl.obolibrary.org/obo/HAO_0001958
	setiferous patch	The patch that differs from the surrounding region by having denser setae.	http://purl.obolibrary.org/obo/HAO_0000936
	speculum	The area that is located dorsally on the mesopleuron, is delimited posteriorly by the mesepimeral ridge and corresponds to the site of origin of t	http://purl.obolibrary.org/obo/HAO_0000944
	spine	The process that lacks non-sclerotised ring at the base.	http://purl.obolibrary.org/obo/HAO_0000949
ste	sternaulus	The line that marks the site of origin of the mesopleuro-basalare and first mesopleuro-mesonotal muscle.	http://purl.obolibrary.org/obo/HAO_0000953
	sternaulus length	The straight anatomical line between the anterior and posterior ends of the sternaulus.	http://purl.obolibrary.org/obo/HAO_0002402
sfc	submedial flange of occipital carina	The flange that is submedial and is on the occipital carina.	http://purl.obolibrary.org/obo/HAO_0002390
stc	subtorular carina	The carina that extends ventrally from the lateral margin of the antennal rim.	http://purl.obolibrary.org/obo/HAO_0002400
	sulcus	The groove that corresponds to a ridge.	http://purl.obolibrary.org/obo/HAO_0000978
	supraclypeal depression	The median impression on the frons that is dorsal to the intertorular carina and is not a scrobe for receiving the scape.	http://purl.obolibrary.org/obo/HAO_0002380
	tegula	The sclerite that is located laterally of the preaxilla and obscures the anterior mesonoto-first axillary articulation and the mesopleuro-second axi	http://purl.obolibrary.org/obo/HAO_0000993
	tergum	The area that is located on the integument and is dorsal of the ventral diaphragm.	http://purl.obolibrary.org/obo/HAO_0001006
	tibia	The leg segment that is proximal to the tarsus and distal to the femur.	http://purl.obolibrary.org/obo/HAO_0001017
	tooth	The projection that is located distally on the mandible.	http://purl.obolibrary.org/obo/HAO_0001019
	torulo-clypeal carina	The carina that is connects the clypeus with the antennal rim.	http://purl.obolibrary.org/obo/HAO_0002399
	transscutal articulation	The sulcus that extends along the transscutal line.	http://purl.obolibrary.org/obo/HAO_0001623
	transverse carina on petiole	The carina that delimits the anterior vertical area of abdominal segment 2.	http://purl.obolibrary.org/obo/HAO_0001029
tfc	transverse frontal carina	The transverse carina that is dorsal to the facial pit and ventral to the anterior ocellus.	http://purl.obolibrary.org/obo/HAO_0002398
	transverse line of the metanotum-prop	The line of the metanoto-propodeo-metapecto-mesopectal complex that connects the humeral sclerite to the third phragma.	http://purl.obolibrary.org/obo/HAO_0002396
	upper face	The area that is located dorsally of the ventral margin of the antennal rim and ventrally of the anterior ocellus medial to the inner margins of the	http://purl.obolibrary.org/obo/HAO_0001044
	ventrolateral invagination of the pron	The invagination that at the ventrolateral edge of the pronotum.	http://purl.obolibrary.org/obo/HAO_0002401
	ventrolateral setiferous patch	The sublateral setiferous patch that is on the frons between the intertorular carina and the facial pit, and is separated from the median setiferous	http://purl.obolibrary.org/obo/HAO_0002382
	ventromedian setiferous patch	The median setiferous patch that is on the frons between the intertorular carina and the facial pit.	http://purl.obolibrary.org/obo/HAO_0002381
	vertex	The area that is delimited by the intersection of the margin of the compound eyes, the interorbital plane, and the anatomical line that is tangenti	http://purl.obolibrary.org/obo/HAO_0001077
	volsella	The anatomical cluster that is composed of the sclerites on the ventral part of the male genitalia that are not connected to the cupula via muscles	http://purl.obolibrary.org/obo/HAO_0001084
	width	The anatomical line that is perpendicular to the longitudinal axis of the anatomical structure and between one side of an object to the opposite s	http://purl.obolibrary.org/obo/HAO_0002375
