# Supplementary material for: Malagasy Conostigmus (Hymenoptera: Ceraphronoidea) and the secret of scutes
Source: PeerJ. 2016 Dec 13;4:e2682. doi: 10.7717/peerj.2682 (PMC5157207; doi:10.7717/peerj.2682)
Supplement: Supplemental Information 8 [file peerj-04-2682-s008.pdf]

Table S4. Semantic statements of natural language phenotypes composed in Protégé 5.0 (http://protege.stanford.edu/) using the OWL Manchester syntax.

Entity	Value	Semantic
Antennal scrobe count	absent	not (has part some antennal scrobe)
Antennal scrobe count	present	has part some antennal scrobe
Anterior mesoscutal width vs posterior mesoscutal width	AscW/PscW=0.9	has part some (anterior mesoscutal width quality measured as some (has measuren in some posterior mesoscutal width)))) a 0.9f])))))
Anterior mesoscutal width vs posterior mesoscutal width	AscW/PscW=0.8-0.9	has part some (anterior mesoscutal width quality measured as some (has measuren in some posterior mesoscutal width)))) a 0.8f , <= 0.9f])))))
Anteromedian projection of the metanoto-propodeo-metapecto-mesopectal complex count	present	has part some anteromedian projection of mesopectal complex
Anteromedian projection of the metanoto-propodeo-metapecto-mesopectal complex count	absent	not (has part some anteromedian projecti mesopectal complex)
Areolate Sculpture on Body Count	absent	not (has part some (anatomical region a
Areolate Sculpture on Body Count	present	(has part some (mesosoma and (bearer frons and (bearer of some areolate)))
Axillular carina count	present	has part some axillular carina
Axillular carina count	absent	not (has part some axillular carina)
Axillular carina shape	left and right carina continuous posteromedially forming a U-shape carina on the mesoscutellar axillar complex	has part some (axillular carina and (not (bearer of some left)))) and (bearer of so
Axillular carina shape	The left and right carina are separated posteromedially	has part some (axillular carina and (bear axillular carina and (bearer of some left
Axillular carina shape	absent	not (has part some axillular carina)

Entity	Value	Semantic
Body length universal	2575 µm	has part some (median anatomical line ar<br>measured as some ((has measurement un<br>measurement value some float [>= 2575.(
Body length universal	2200 µm	has part some (median anatomical line ar<br>measured as some ((has measurement un<br>measurement value some float [>= 2200.(
Body length universal	1500-2700 µm	has part some (median anatomical line ar<br>measured as some ((has measurement un<br>measurement value some (float [>= 1500
Body length universal	1.4-1.7 mm	has part some (median anatomical line ar<br>measured as some ((has measurement un<br>measurement value some (float [>= 1.0f]
Body length universal	2.0-2.3 mm	has part some (median anatomical line ar<br>measured as some ((has measurement un<br>measurement value some (float [>= 2.0f]
Body length universal	1150-2300 µm	has part some (median anatomical line ar<br>measured as some ((has measurement un<br>measurement value some (float [>= 1150
Body length universal	2450-3125 µm	has part some (median anatomical line ar<br>measured as some ((has measurement un<br>measurement value some (float [>= 2450
Body length universal	2.6-2.7 mm	has part some (median anatomical line ar<br>measured as some ((has measurement un<br>measurement value some (float [>= 2.6f]
Body length universal	2100-2600 µm	has part some (median anatomical line ar<br>measured as some ((has measurement un<br>measurement value some (float [>= 2100
Body length universal	5.3-6.6 mm	has part some (median anatomical line ar<br>measured as some ((has measurement un<br>measurement value some (float [>= 5.3f]
Body length universal	1750-2000 µm	has part some (median anatomical line ar<br>measured as some ((has measurement un<br>measurement value some (float [>= 1750
Body length universal	6.5-6.8 mm	has part some (median anatomical line ar<br>measured as some ((has measurement un<br>measurement value some (float [>= 6.5f]

Entity	Value	Semantic
Body length universal	1750-2450 µm	has part some (median anatomical line an measured as some ((has measurement un measurement value some (float [>= 1750
Body length universal	1650-1875 µm	has part some (median anatomical line an measured as some ((has measurement un measurement value some (float [>= 1650
Body length universal	4.6-8.0 mm	has part some (median anatomical line an measured as some ((has measurement un measurement value some (float [>= 4.6f]
Body length universal	2000-3450 µm	has part some (median anatomical line an measured as some ((has measurement un measurement value some (float [>= 2000
Body length universal	2.4-2.6 mm	has part some (body and (bearer of some some ((has measurement unit label value some (float [>= 2.4f] and float [<= 2.6f])
Body length universal	2.4-3.0 mm	has part some (median anatomical line an measured as some ((has measurement un measurement value some (float [>= 2.4f]
Body length universal	2325-2500 µm	has part some (median anatomical line an measured as some ((has measurement un measurement value some (float [>= 2325
Body length universal	7.0-7.8 mm	has part some (median anatomical line an measured as some ((has measurement un measurement value some (float [>= 7.0f]
Body length universal	1.8-1.9 mm	has part some (median anatomical line an measured as some ((has measurement un measurement value some (float [>= 1.8f]
Body length universal	5.5-9.1 mm	has part some (median anatomical line an measured as some ((has measurement un measurement value some (float [>= 5.5f]
Body length universal	1270-1300 µm	has part some (median anatomical line an measured as some ((has measurement un measurement value some (float [>= 1270
Cephalic size (csb)	Mean: 4.8-6.0	has part some (absolute cephalic size and measured as some ((has measurement un measurement value some (float [>= 4.8f]

Entity	Value	Semantic
Cephalic size (csb)	Mean: 2.0-4.5	has part some (absolute cephalic size and measured as some ((has measurement un measurement value some (float [>= 2.0f]
Color hue pattern	Scape, hind leg except metacoxa ochre; fore and hind legs, distal metacoxa yellow; rest of body brown	((not (scape)) and (part_of some (body part some (fore leg and (bearer of some and (bearer of some yellow))) and (has ochre))) and (has part some (metafemur
Color hue pattern	Antenna except pedicel and scape, cranium, mesosoma except fore and middle legs and distal region of metacoxa, and metasoma brown; fore and middle legs. tegula, pedicel, scape, maxillary palp, and labial palp yellow	((not (pedicel)) and (part_of some (ant has part some (not (scape)))) and (has p (metacoxa and (bearer of some brown)) bearer of some brown))) and (has part so yellow))) and (has part some (labial pal part some (maxillary palp and (bearer of mesosoma and (bearer of some brown)) bearer of some brown))) and (has part so yellow))) and (has part some (pedicel ar part some (scape and (bearer of some ye bearer of some yellow)))
Color hue pattern	Legs except proximal region of metacoxa and distal 2/3 of metafemur, mouthparts, scape and F1 orange; rest of body brown	((not (mouthparts)) and (not (scape)) ar some (body and (bearer of some brown and (part_of some (metacoxa and (beare (distal region and (part_of some (metafe (has part some (mouthparts and (bearer scape and (bearer of some orange))) and bearer of some orange))) and (has part s some (metacoxa and (bearer of some ora region and (part_of some (metafemur ar
Color hue pattern	Cranium, mesosoma brown; antenna, legs except brown metacoxa, metasoma ochre	((part_of some (leg and (bearer of some metacoxa)))) and (has part some (anten part some (cranium and (bearer of some and (bearer of some brown))) and (has p ochre)))
Color hue pattern	Antenna except pedicel, cranium, mesosoma except fore and middle legs and distal region of metacoxa, and metasoma brown; fore and middle legs, tegula, pedicel, proximal region of metacoxa, maxillary palp, and labial palp yellow	((part_of some (antenna and (bearer of pedicel)))) and (has part some (proxima and (bearer of some yellow))))) and (ha some (metacoxa and (bearer of some bro and (bearer of some brown))) and (has p yellow))) and (has part some (labial pal part some (maxillary palp and (bearer of mesosoma and (bearer of some brown)) bearer of some brown))) and (has part so yellow))) and (has part some (pedicel ar part some (scape and (bearer of some ye

Entity	Value	Semantic
		bearer of some yellow)))
Color hue pattern	scape, pedicel, F1-3, head, anterior mesosoma ochre, F4-F9, posterior mesosoma, metasoma brown, legs except darker proximal regions of meso and metacoxae yellow	(has part some (cranium and (bearer of and ((proximal region and (part_of some and (part_of some mid coxa)))) and (be (mesosoma and (has part some (anterio and (has part some (metacoxa and (has of some brown))))) and (has part some (region and (bearer of some brown))))) a some ochre))) and (has part some (scap part some (first flagellomere and (beare second flagellomere and (bearer of some flagellomere and (bearer of some brown and (bearer of some ochre))) and (has p of some brown))) and (has part some (s brown))) and (has part some (seventh fl and (has part some (eighth flagellomere part some (ninth flagellomere and (beare
Color hue pattern	Antenna except pedicel and scape, cranium, mesosoma except fore and middle legs and distal region of metacoxa, and metasoma brown; fore and middle legs, tegula, pedicel, scape, proximal part of metacoxa, palpus maxillaris, and palpus labialis yellow	((not (pedicel)) and (part_of some (ant has part some (not (scape)))) and (has p some (metacoxa and (bearer of some ye region and (part_of some (metacoxa and part some (cranium and (bearer of some and (bearer of some yellow))) and (has some yellow))) and (has part some (ma and (has part some (mesosoma and (bea (metasoma and (bearer of some brown) bearer of some yellow))) and (has part s yellow))) and (has part some (scape and some (tegula and (bearer of some yellow
Color hue pattern	F3-8, cranium, mandible, metasoma, tegula brown; legs, except brown proximal region of metacoxa and distal region of metafemur, scape, pedicel, F1-4 yellow	((not (distal region and (part_of some m bearer of some yellow))) and (has part s some metacoxa))))) and (has part some (and (has part some (mandible and (bear metasoma and (bearer of some brown))) of some yellow))) and (has part some (s has part some (tegula and (bearer of som flagellomere and (bearer of some yellow flagellomere and (bearer of some yellow and (bearer of some brown))) and (has p of some yellow))) and (has part some (f brown))) and (has part some (sixth flag and (has part some (seventh flagellomer part some (eighth flagellomere and (bea
Color hue pattern	Dark brown except pedicel, proximal 1/5th of	((not (fore leg)) and (not (mid leg)) and scape))) and (part_of some (body and (

Entity	Value	Semantic
	scape, fore and middle leg, mandible ochre/yellowish	part some (not (pedicel)))) and (has par[illegible] some (scape and (bearer of some yellow[illegible] bearer of some ochre))) and (has part so[illegible] ochre))) and (has part some (mid leg an[illegible] some (pedicel and (bearer of some ochre[illegible]
Color hue pattern	Legs except proximal region of metacoxa and distal region of metafemur, mouthparts yellow; rest of body ochre	((not (mouthparts)) and (part_of some ([illegible] has part some (proximal region and (par[illegible] ochre))))) and (has part some (distal reg[illegible] bearer of some ochre))))) and (has part s[illegible] yellow))) and (has part some (not (prox[illegible] and (bearer of some yellow)))))) and (h[illegible] part_of some (metafemur and (bearer of[illegible]
Color hue pattern	Cranium, mesosoma except legs and metasoma except gonostipes and volsella ochre; antenna, legs, mandible, gonostipes and volsella yellow	(has part some (antenna and (bearer of [illegible] cranium and (bearer of some ochre))) an[illegible] of some yellow))) and (has part some ([illegible] has part some (mandible and (bearer of [illegible] mesosoma and (bearer of some ochre))) [illegible] bearer of some ochre))) and (has part so[illegible] yellow)))
Color hue pattern	Scape, F4-8, cranium, mandible, metasoma, tegula brown; legs, except brown proximal region of metacoxa and distal region of metafemur, pedicel, F1-3 yellow	(has part some (proximal region and (pa[illegible] some brown))))) and (has part some (di[illegible] and (bearer of some brown))))) and (has[illegible] brown))) and (has part some (mandible [illegible] part some (metasoma and (bearer of son[illegible] and (bearer of some yellow))) and (has [illegible] brown))) and (has part some (tegula and[illegible] some (first flagellomere and (bearer of s[illegible] second flagellomere and (bearer of some[illegible] flagellomere and (bearer of some brown [illegible] and (bearer of some yellow))) and (has [illegible] bearer of some brown))) and (has part so[illegible] some brown))) and (has part some (seve[illegible] brown))) and (has part some (eighth fla[illegible] and (has part some (not (proximal region[illegible] bearer of some yellow)))))) and (has par[illegible] some (metafemur and (bearer of some y[illegible]
Color hue pattern	Legs except proximal region of metacoxa and distal 2/3 of metafemur, mouthparts, distal 1/5 of scape, yellow; rest of body brown	((not (mouthparts)) and (part_of some ([illegible] (has part some (proximal region and (pa[illegible] some brown))))) and (has part some (di[illegible] bearer of some yellow))))) and (has part[illegible] metafemur and (bearer of some brown))[illegible] bearer of some yellow))) and (has part s[illegible] some (metacoxa and (bearer of some ye[illegible] region and (part_of some (scape and (b[illegible] some (not (distal region and (part_of so[illegible]

Entity	Value	Semantic
		yellow))))))
Color hue pattern	Cranium, mandible, mesosoma excluding front and proximal middle tibia, metasoma, antenna excluding distal scape and pedicel brown; distal scape, pedicel, protibia and proximal mesotibia ochre	(has part some (proximal region and (pa some ochre))))) and (has part some (dis bearer of some ochre))))) and (has part s brown))) and (has part some (fore tibia part some (mandible and (bearer of som mesosoma and (bearer of some brown)) bearer of some brown))) and (has part so ochre))) and (has part some ((not (pedic region and (part_of some (scape and (be some (antenna and (bearer of some brow
Color hue pattern	Cranium black; mesosoma, metasoma, F4-F9 brown; rest of antenna, legs and mandible ochre	(has part some (cranium and (bearer of and (bearer of some ochre))) and (has p ochre))) and (has part some (mesosoma part some (metasoma and (bearer of son flagellomere and (bearer of some ochre) flagellomere and (bearer of some ochre) and (bearer of some brown))) and (has p of some ochre))) and (has part some (fo brown))) and (has part some (sixth flage and (has part some (seventh flagellomer part some (eighth flagellomere and (bea ninth flagellomere and (bearer of some b
Color hue pattern	Distal part of scape, pedicel, F1-3 ochre; legs except proximal metacoxa yellow; rest of body brown	((not (pedicel)) and (not (first flagellom and (not (third flagellomere)) and (part_ brown))) and (has part some (not (distal (has part some (distal region and (part_))))) and (has part some (pedicel and (b (first flagellomere and (bearer of some o flagellomere and (bearer of some ochre) and (bearer of some ochre))) and (has p part some metacoxa))) and (part_of som
Color hue pattern	Antenna, legs, mouthparts ochre; rest of body dark brown	(has part some (antenna and (bearer of s and (not (antenna)) and (not (leg)) and dark brown))) and (has part some (leg a some (mouthparts and (bearer of some o
Color hue pattern	Cranium brown; mesosoma except legs, metasoma ochre; F4-F9 brown; rest of antenna ochre, legs yellow	(has part some (cranium and (bearer of and (bearer of some yellow))) and (has some ochre))) and (has part some (meta has part some (first flagellomere and (be (second flagellomere and (bearer of som flagellomere and (bearer of some brown and (bearer of some ochre))) and (has p of some brown))) and (has part some (s

Entity	Value	Semantic
		brown))) and (has part some (seventh fl and (has part some (eighth flagellomere part some (ninth flagellomere and (beare
Color hue pattern	Distal half of scape, pedicel, fore leg and middle leg yellow; proximal part of scape, flagellum, mesosoma except front and middle leg, metasoma brown	(has part some (proximal region and (pa brown))))) and (has part some (distal re bearer of some yellow))))) and (has part brown))) and (has part some (fore leg a part some (mesosoma and (bearer of so metasoma and (bearer of some brown))) of some yellow))) and (has part some (p
Color hue pattern	Antenna except pedicel, cranium, mesosoma except fore and middle legs and metasoma brown; fore and middle legs, tegula, pedicel, maxillary palp and labial palp yellow	((part_of some (antenna and (bearer of pedicel)))) and (has part some (cranium part some (fore leg and (bearer of some and (bearer of some yellow))) and (has some yellow))) and (has part some (mes (has part some (metasoma and (bearer o mid leg and (bearer of some yellow))) a some yellow))) and (has part some (sca has part some (tegula and (bearer of som
Color hue pattern	Legs except proximal region of metacoxa and distal 2/3 of metafemur, mouthparts, distal part of scape, pedicel and F1-F4 yellow; rest of body brown	((not (mouthparts)) and (not (pedicel)) second flagellomere)) and (not (third fla)) and (part_of some (body and (bearer proximal region and (part_of some (met (has part some (distal region and (part_ yellow))))) and (has part some (distal re bearer of some brown))))) and (has part yellow))) and (has part some (first flage and (has part some (second flagellomere part some (third flagellomere and (beare fourth flagellomere and (bearer of some proximal region and (part_of some (met and (has part some (not (distal region an some yellow)))))) and (has part some (n metafemur and (bearer of some yellow))
Color hue pattern	F1-8, cranium, mandible, metasoma, tegula brown; legs, scape, pedicel yellow	(has part some (cranium and (bearer of and (bearer of some yellow))) and (has brown))) and (has part some (metasoma part some (pedicel and (bearer of some bearer of some yellow))) and (has part s brown))) and (has part some (first flage and (has part some (second flagellomere part some (fifth flagellomere and (beare third flagellomere and (bearer of some br flagellomere and (bearer of some brown and (bearer of some brown))) and (has

Entity	Value	Semantic
		bearer of some brown))) and (has part so<br>some brown)))
Color hue pattern female	Cranium except supraclypeal depression and mesosoma except posteroventral region metallic brown/purple; F9, distal and proximal region of scape, supraclypeal depression, abdomen, dorsal proximal regions of femur and tibia brown; F1-F5, pedicel, scape exce	(has part some (abdomen and (bearer of<br>metasomal segment and (bearer of some<br>(bearer of some white))) and (has part s<br>some white))) and (has part some (fifth<br>white))) and (has part some (third flage<br>(has part some (fourth flagellomere and<br>some ((not (postero-ventral region)) and<br>of some (iridescent and (bearer of some<br>distal region)) and (not (ventral region)<br>some brown))))) and (has part some ((no<br>scape and (bearer of some white))))) and<br>depression)) and (part_of some (craniu<br>bearer of some brown)))))))
Color hue pattern male	Cranium, mesosoma, F1-9, pedicel, distal region of hind femur, abdomen brown; scape, forelegs and midlegs, tibia of hind leg yellow; Hind coxa and petiole neck white	(has part some (distal region and (part_<br>brown))))) and (has part some (abdome<br>part some (first metasomal segment and<br>some (cranium and (bearer of some brow<br>bearer of some yellow))) and (has part s<br>brown))) and (has part some (metacoxa<br>part some (mid leg and (bearer of some<br>and (bearer of some brown))) and (has p<br>of some brown))) and (has part some (s<br>brown))) and (has part some (fifth flage<br>and (has part some (third flagellomere a<br>part some (fourth flagellomere and (bear<br>sixth flagellomere and (bearer of some b<br>flagellomere and (bearer of some brown<br>flagellomere and (bearer of some brown<br>flagellomere and (bearer of some brown<br>)) and (part_of some (hind leg and (bea
Color intensity pattern	Legs lighter than scape and mandible	has part some (leg and (bearer of some (<br>increased_in_magnitude_relative_to som<br>inheres in some scape)))))))
Color intensity pattern	flagellum, tibiae and tarsi lighter than scape, pedicel, mandible, tegula, coxae and femora	has part some (flagellum and tarsomere a<br>brightness and (increased_in_magnitude<br>coxa and femur and mandible and pedicel<br>scape)))))))
Color intensity pattern	flagellomeres and their branches darker than scape and pedicel. Scape and pedicel same as legs	has part some (flagellomere and (bearer<br>decreased_in_magnitude_relative_to som<br>inheres in some scape))))))

Entity	Value	Semantic
Color intensity pattern	metasoma and mandible lighter than mesosoma	has part some (mandible and metasoma a (increased_in_magnitude_relative_to som mesosoma))))))
Color intensity pattern	metasoma lighter than mesosoma and cranium	has part some (metasoma and (bearer of increased_in_magnitude_relative_to som inheres in some mesosoma)))))))
Color intensity pattern	ventral region of cranium is lighter than dorsal region of cranium	has part some (ventral region and (part_ color brightness and (increased_in_magn and (inheres in some (dorsal region and
Color intensity pattern	front and middle leg lighter than distal half of scape, pedicel and tegula; cranium, distal region of flagellum, mesosoma except legs and petiole neck darker than proximal region of flagellum, hind leg and metasoma posterior to petiole neck and mesosoma d	(has part some (cranium and mesosoma flagellum)) and (bearer of some (color l increased_in_magnitude_relative_to som distal region and (part_of some flagellum and (has part some (fore leg and mid leg and (increased_in_magnitude_relative_to some (pedicel and tegula and (distal reg
Color intensity pattern	distal scape, legs except hind coxa lighter than metasoma	(has part some (distal region and (part_ and (has part some (not (metacoxa))))) (increased_in_magnitude_relative_to som metasoma)))))
Cupula length vs. gonostyle-volsella complex length	cupula less than 1/2 the length of gonostyle-volsella complex in lateral view	has part some (cupula and (bearer of sor some (has measurement unit label some gonostipes/volsella complex)))) and (has 0.5f])))))
Cupula length vs. gonostyle-volsella complex length	cupula as long as gonostipes in lateral view	has part some (cupula and (has part som bearer of some (length and (similar_in_r inheres in some (proximodistal anatomic gonostipes/volsella complex))))))))))
Distal end of dorsomedian conjunctiva of the gonostyle-volsella complex shape	acute	has part some (dorsomedian conjunctiva has part some (distal margin and (bearer
Distal end of dorsomedian conjunctiva of the gonostyle-volsella complex shape	blunt	has part some (dorsomedian conjunctiva has part some (distal margin and (bearer
Distal margin of harpe in lateral view: shape	blunt	has part some (harpe and (has part some blunt))))

Entity	Value	Semantic
Distal margin of harpe in lateral view: shape	acute	has part some (harpe and (has part some [illegible] acute))))
Distal margin of male S9 shape	concave	has part some (abdominal sternum 9 and [illegible] bearer of some concave))))
Distal margin of male S9 shape	convex	has part some (abdominal sternum 9 and [illegible] bearer of some convex))))
Distal margin of male S9 shape	straight	has part some (abdominal sternum 9 and [illegible] bearer of some straight))))
Distal projection of parossiculus presence	absent	not (has part some distal projection of th[illegible]
Distal projection of parossiculus presence	present	has part some distal projection of the paro[illegible]
Distal projection of the penisvalva count	absent	not (has part some distal projection of th[illegible]
Distal projection of the penisvalva count	present	has part some distal projection of the pen[illegible]
Distodorsal margin of cupula shape	straight	has part some (cupula and (has part som[illegible] margin and (bearer of some straight))))[illegible]
Distodorsal margin of cupula shape	concave	has part some (cupula and (has part som[illegible] margin and (bearer of some concave))))[illegible]
Distodorsal setae of sensillar ring of harpe length vs. harpe width in lateral view	>2	has part some (sensillar ring of harpe an[illegible] part some (distal margin and (has part s[illegible] some (proximodistal anatomical line and [illegible] measured as some (has measurement uni[illegible] harpe width)))) and (has measurement v[illegible]
Distodorsal setae of sensillar ring of harpe length vs. harpe width in lateral view	setae as long or shorter than harpe width	has part some (sensillar ring of harpe an[illegible] part some (distal margin and (has part s[illegible] some (proximodistal anatomical line and [illegible] measured as some (has measurement uni[illegible] harpe width)))) and (has measurement v[illegible]
Distodorsal setae of sensillar ring of harpe length vs. harpe width in lateral view	setae longer than harpe width	has part some (sensillar ring of harpe an[illegible] part some (distal margin and (has part s[illegible] some (proximodistal anatomical line and [illegible] measured as some (has measurement uni[illegible] harpe width)))) and (has measurement v[illegible]
Distodorsal setae of sensillar ring of harpe orientation	distomedially	has part some (sensillar ring of harpe an[illegible] part some (distal margin and (has part s[illegible]

Entity	Value	Semantic
		some (medial orientation and (bearer of
Distodorsal setae of sensillar ring of harpe orientation	medially	has part some (sensillar ring of harpe and part some (distal margin and (has part so some medial orientation))))))))
Distodorsal setae of sensillar ring of harpe orientation	dorsally	has part some (sensillar ring of harpe and part some (distal margin and (has part so some dorsal orientation))))))))
Distoventral submedian corner of cupula count	absent	not (has part some proximolateral projec
Distoventral submedian corner of cupula count	present	has part some proximolateral projection o
Dorsal apodeme of the penisvalva: presence	absent	not (has part some dorsal apodeme of pe
Dorsal apodeme of the penisvalva: presence	present	has part some dorsal apodeme of penisval
Dorsal margin of occipital carina vs. dorsal margin of lateral ocellus in lateral view	occipital carina is ventral to lateral ocellus in lateral view	has part some (occipital carina and (has some dorsal margin) and (ventral_to som lateral side and (part_of some lateral oce
Dorsal margin of occipital carina vs. dorsal margin of lateral ocellus in lateral view	occipital carina is dorsal to lateral ocellus in lateral view	has part some (occipital carina and (has some dorsal margin) and (dorsal_to som lateral side and (part_of some lateral oce
Dorsomedial conjunctiva of the gonostyle/volsella complex count	absent	not (has part some dorsomedian conjunc
Dorsomedial conjunctiva of the gonostyle/volsella complex count	present	has part some dorsomedian conjunctiva o
Dorsomedial conjunctiva of the gonostyle/volsella complex lenght relative to length of gonostyle/volsella complex	dorsomedial conjunctiva extending 2/3 of length of gonostyle/volella complex in dorsal view	has part some (dorsomedian conjunctiva bearer of some (length and ((is quality m label some (length and (inheres in some measurement value some float [>= 0.66f]
Dorsomedial conjunctiva of the gonostyle/volsella complex lenght relative to length of gonostyle/volsella complex	dorsomedial conjunctiva not extending 2/3 of length of gonostyle/volsella complex in dorsal view	has part some (dorsomedian conjunctiva bearer of some (length and ((is quality m label some (length and (inheres in some measurement value some float [< 0.66f])
Epicnemial carina presence	present only medially	has part some (epicnemial carina and (no part some medial region))

Entity	Value	Semantic
Epicnemial carina presence	complete	has part some (epicnemial carina and (h some medial region))
Epicnemial carina presence	interupted medially	has part some (epicnemial carina and (no
Epicnemial carina presence	present only laterally	has part some (epicnemial carina and (no has part some lateral region))
Epicnemial carina presence	absent	not (has part some epicnemial carina)
Epicnemium posterior margin shape	anterior discrimenal pit absent; epicnemial carina straight	(not (has part some anterior discrimenal carina and (not (bearer of some curved))
Epicnemium posterior margin shape	anterior discrimenal pit present; epicnemial carina curved	(has part some anterior discrimenal pit) and (bearer of some curved)))
Epicnemium posterior margin shape	anterior discrimenal pit absent, epicnemial carina interrupted medially	(not (has part some anterior discrimenal region and (part_of some epicnemial car
Epicnemium posterior margin shape	anterior discrimenal pit absent, epicnemial carina curved	(not (has part some anterior discrimenal carina and (bearer of some curved)))
Facial pit count	facial process present	has part some facial process
Facial pit count	facial pit present	has part some facial pit
Facial pit count	median facial keel present	has part some median facial keel
Facial pit count	no external corresponding structure present	(not (has part some facial pit)) or (not ((has part some facial process))
Female F9 length	F8+F7<F7+F8+F6	has part some ((seventh flagellomere and anatomical line and (bearer of some leng part some (proximodistal anatomical line decreased_in_magnitude_relative_to som proximodistal anatomical line and (part_ length and (inheres in some (proximodis eighth flagellomere)))))))
Female F9 length	F9=F7+F8	has part some (ninth flagellomere and (h line and (bearer of some (length and (si length and (inheres in some (proximodis seventh flagellomere)))) and (length and

Entity	Value	Semantic
		anatomical line and (part_of some eighth
Female F9 length	F9<F8+F7	has part some (ninth flagellomere and (h line and (bearer of some (length and (de ((length and (inheres in some (proximod seventh flagellomere)))) and (length and anatomical line and (part_of some eighth
Female F9 length	F9=F8	has part some (ninth flagellomere and (h line and (bearer of some (length and (si length and (inheres in some (proximodis eighth flagellomere))))))))))
Female F9 length	F9=F6+F7+F8	has part some (ninth flagellomere and (h line and (bearer of some (length and (si length and (inheres in some (proximodis sixth flagellomere)))) and (length and (i line and (part_of some seventh flagellom (proximodistal anatomical line and (part
Female first flagellomere length vs. pedicel	0.9-1.0	has part some (first flagellomere and (be measured as some (has measurement uni pedicel)))) and (has measurement value
Female first flagellomere length vs. pedicel	0.7	has part some (first flagellomere and (ha line and (bearer of some (length and ((i measurement unit label some (length and some proximodistal anatomical line))))) [>= 0.7f]))))))
Female first flagellomere length vs. pedicel	0.9	has part some (first flagellomere and (ha line and (bearer of some (length and ((i measurement unit label some (length and some proximodistal anatomical line))))) [>= 0.9f]))))))
Female first flagellomere length vs. pedicel	0.8-1.16	has part some (first flagellomere and (be measured as some (has measurement uni pedicel)))) and (has measurement value
Female first flagellomere length vs. pedicel	0.8-0.9	has part some (first flagellomere and (be measured as some (has measurement uni pedicel)))) and (has measurement value
Female first flagellomere length vs. pedicel	F1 as long as pedicel (1.0-1.1)	has part some (first flagellomere and (be measured as some (has measurement uni pedicel)))) and (has measurement value

Entity	Value	Semantic
Female first flagellomere length vs. pedicel	0.8-1.2	has part some (first flagellomere and (be<br>measured as some (has measurement uni<br>pedicel)))) and (has measurement value
Female first flagellomere length vs. pedicel	1.0	has part some (first flagellomere and (ha<br>line and (bearer of some (length and ((i<br>measurement unit label some (length and<br>some proximodistal anatomical line))))) [>= 1.0f])))))))
Female first flagellomere length vs. pedicel	F1 is 1.5 times as long as pedicel	has part some (first flagellomere and (ha<br>line and (bearer of some (length and ((i<br>measurement unit label some (length and<br>some proximodistal anatomical line))))) [>= 1.5f])))))))
Female first flagellomere length vs. pedicel	pedicel longer than F1	has part some (pedicel and (has part som<br>increased_in_magnitude_relative_to som<br>proximodistal anatomical line and (part_
Female first flagellomere length vs. pedicel	0.85	has part some (first flagellomere and (ha<br>line and (bearer of some (length and ((i<br>measurement unit label some (length and<br>some proximodistal anatomical line))))) [>= 0.85f])))))))
Female first flagellomere length vs. pedicel	1.4	has part some (first flagellomere and (ha<br>line and (bearer of some (length and ((i<br>measurement unit label some (length and<br>some proximodistal anatomical line))))) [>= 1.4f])))))))
Female first flagellomere length vs. pedicel	1.09	has part some (first flagellomere and (ha<br>line and (bearer of some (length and ((i<br>measurement unit label some (length and<br>some proximodistal anatomical line))))) [>= 1.09f])))))))
Female ocular ocellar line (OOL):posterior ocellar line (POL):lateral ocellar line(LOL)	0.85:0.85:1.00	(has part some (ocular ocellar line and (<br>measured as some (has measurement uni<br>lateral ocellar line)))) and (has measurem<br>has part some (posterior ocellar line and<br>measured as some (has measurement uni<br>lateral ocellar line)))) and (has measurem
Female ocular ocellar line (OOL):posterior ocellar line (POL):lateral ocellar line(LOL)	1.4:1.6-1.7:1.0	(has part some (ocular ocellar line and (<br>measured as some (has measurement uni<br>lateral ocellar line)))) and (has measurem<br>has part some (posterior ocellar line and

Entity	Value	Semantic
		measured as some (has measurement uni lateral ocellar line)))) and (has measuren 1.7f]))))))
Female ocular ocellar line (OOL):posterior ocellar line (POL):lateral ocellar line(LOL)	2.5-3.0:1.9-2.0:1.0	(has part some (ocular ocellar line and (measured as some (has measurement uni lateral ocellar line)))) and (has measuren 3.0f])))))) and (has part some (posterior and ((is quality measured as some (has r inheres in some lateral ocellar line)))) an 1.9f , <= 2.0f]))))))
Female ocular ocellar line (OOL):posterior ocellar line (POL):lateral ocellar line(LOL)	1.7-2.3:1.7-1.8:1.0	(has part some (ocular ocellar line and (measured as some (has measurement uni lateral ocellar line)))) and (has measuren 2.3f])))))) and (has part some (posterior and ((is quality measured as some (has r inheres in some lateral ocellar line)))) an 1.7f , <= 1.8f]))))))
Female ocular ocellar line (OOL):posterior ocellar line (POL):lateral ocellar line(LOL)	1.0:1.2:1.0	(has part some (ocular ocellar line and (measured as some (has measurement uni lateral ocellar line)))) and (has measuren has part some (posterior ocellar line and measured as some (has measurement uni lateral ocellar line)))) and (has measuren
Female ocular ocellar line (OOL):posterior ocellar line (POL):lateral ocellar line(LOL)	1.5-2.1:1.2-1.4:1.0	(has part some (ocular ocellar line and (measured as some (has measurement uni lateral ocellar line)))) and (has measuren 2.1f])))))) and (has part some (posterior and ((is quality measured as some (has r inheres in some lateral ocellar line)))) an 1.2f , <= 1.4f]))))))
Female ocular ocellar line (OOL):posterior ocellar line (POL):lateral ocellar line(LOL)	1.4:1.0-1.2:1.0	(has part some (ocular ocellar line and (measured as some (has measurement uni lateral ocellar line)))) and (has measuren has part some (posterior ocellar line and measured as some (has measurement uni lateral ocellar line)))) and (has measuren 1.2f]))))))
Female ocular ocellar line (OOL):posterior ocellar line (POL):lateral ocellar line(LOL)	1.0-1.1:1.4:1.0	(has part some (ocular ocellar line and (measured as some (has measurement uni lateral ocellar line)))) and (has measuren 1.1f])))))) and (has part some (posterior and ((is quality measured as some (has r inheres in some lateral ocellar line)))) an

Entity	Value	Semantic
		1.0f]))))))
Female ocular ocellar line (OOL):posterior ocellar line (POL):lateral ocellar line(LOL)	1.2-1.3:1.0:1.0	(has part some (ocular ocellar line and (measured as some (has measurement uni lateral ocellar line)))) and (has measurer 1.3f])))))) and (has part some (posterior and ((is quality measured as some (has r inheres in some lateral ocellar line)))) an 1.0f]))))))
Female ocular ocellar line (OOL):posterior ocellar line (POL):lateral ocellar line(LOL)	3.4:2.1-2.2:1.0	(has part some (ocular ocellar line and (measured as some (has measurement uni lateral ocellar line)))) and (has measurer has part some (posterior ocellar line and measured as some (has measurement uni lateral ocellar line)))) and (has measurer 2.2f]))))))
Female OOL vs. LOL	OOL 1.0-1.2 x as long as LOL	has part some (ocular ocellar line and (b measured as some (has measurement uni lateral ocellar line)))) and (has measurer 1.2f])))))
Female OOL vs. LOL	OOL 1.9-2.1 x as long as LOL	has part some (ocular ocellar line and (b measured as some (has measurement uni lateral ocellar line)))) and (has measurer 2.1f])))))
Female OOL vs. LOL	2: OOL 1.5-2.5 x as long as LOL	has part some (ocular ocellar line and (b measured as some (has measurement uni lateral ocellar line)))) and (has measurer 2.5f])))))
Female OOL vs. LOL	OOL 0.625-0.75 as long as LOL	has part some (ocular ocellar line and (b measured as some (has measurement uni lateral ocellar line)))) and (has measurer 0.75f])))))
Harpe length	harpe as long as gonostipes in lateral view	has part some (harpe and (has part some bearer of some (length and (similar_in_r inheres in some (proximodistal anatomic gonostipes))))))))))
Harpe length	harpe shorter than gonostipes in lateral view	has part some (harpe and (has part some bearer of some (length and (decreased_i and (inheres in some (proximodistal ana))))))))))

Entity	Value	Semantic
Head height (lateral view) vs eye height (anterior view)	HH/EH=1.4-1.8	has part some (head height and (bearer o as some (has measurement unit label son height)))) and (has measurement value s
Head height (lateral view) vs eye height (anterior view)	HH:EHf=1.0-2.0	has part some (head height and (bearer o as some (has measurement unit label son height)))) and (has measurement value s
Head height vs. head length	HH:HL=1.5-2.0	has part some (head height and (bearer o as some (has measurement unit label son length)))) and (has measurement value s
Head height vs. head length	HH/HL=1.4-1.8	has part some (head height and (bearer o as some (has measurement unit label son length)))) and (has measurement value s
Head height vs. head length	HH:HL=1.0-1.5	has part some (head height and (bearer o as some (has measurement unit label son length)))) and (has measurement value s
Head width vs. head height	HW/HH=1.0-1.5	has part some (head width and (bearer o as some (has measurement unit label son height)))) and (has measurement value s
Head width vs. head height	HW/HH=1.2-1.4	has part some (head width and (bearer o as some (has measurement unit label son height)))) and (has measurement value s
Head width vs. interorbital space	HW/IOS=1.8-2.0	has part some (head width and (bearer o as some (has measurement unit label son interorbital space)))) and (has measurem 2.0f]))))
Head width vs. interorbital space	HW/IOS=2.0-2.5	has part some (head width and (bearer o as some (has measurement unit label son interorbital space)))) and (has measurem 2.5f]))))
Head width vs. interorbital space (HW/IOS) Female	2.2	has part some (head width and (bearer o as some (has measurement unit label son interorbital space)))) and (has measurem
Head width vs. interorbital space (HW/IOS) Female	2.3	has part some (head width and (bearer o as some (has measurement unit label son interorbital space)))) and (has measurem
Head width vs. interorbital space (HW/IOS) Female	1.7-1.8	has part some (head width and (bearer o as some (has measurement unit label son

Entity	Value	Semantic
		interorbital space)))) and (has measurem 1.8f]))))
Head width vs. interorbital space (HW/IOS) Female	2.4	has part some (head width and (bearer o as some (has measurement unit label son interorbital space)))) and (has measurem
Head width vs. interorbital space (HW/IOS) Female	2.3-2.6	has part some (head width and (bearer o as some (has measurement unit label son interorbital space)))) and (has measurem 2.6f]))))
Head width vs. interorbital space (HW/IOS) Female	1.6-1.7	has part some (head width and (bearer o as some (has measurement unit label son interorbital space)))) and (has measurem 1.7f]))))
Head width vs. interorbital space (HW/IOS) Female	2.3-2.7	has part some (head width and (bearer o as some (has measurement unit label son interorbital space)))) and (has measurem 2.7f]))))
Head width vs. interorbital space (HW/IOS) Female	2.3-2.4	has part some (head width and (bearer o as some (has measurement unit label son interorbital space)))) and (has measurem 2.4f]))))
Head width vs. interorbital space (HW/IOS) Female	2.0-2.2	has part some (head width and (bearer o as some (has measurement unit label son interorbital space)))) and (has measurem 2.2f]))))
Head width vs. interorbital space (HW/IOS) Female	2.0-2.1	has part some (head width and (bearer o as some (has measurement unit label son interorbital space)))) and (has measurem 2.1f]))))
Head width vs. interorbital space (HW/IOS) Female	2.65	has part some (head width and (bearer o as some (has measurement unit label son interorbital space)))) and (has measurem
Head width vs. interorbital space (HW/IOS) Male	2.1	has part some (head and (bearer of some some (has measurement unit label some space)))) and (has measurement value so
Head width vs. interorbital space (HW/IOS) Male	1.8-1.9	has part some (head and (bearer of some some (has measurement unit label some

Entity	Value	Semantic
		space)))) and (has measurement value so
Head width vs. interorbital space (HW/IOS) Male	1.6-1.9	has part some (head and (bearer of some some (has measurement unit label some space)))) and (has measurement value so
Head width vs. interorbital space (HW/IOS) Male	1.9-2.0	has part some (head and (bearer of some some (has measurement unit label some space)))) and (has measurement value so
Head width vs. interorbital space (HW/IOS) Male	1.6-1.7	has part some (head and (bearer of some some (has measurement unit label some space)))) and (has measurement value so
Head width vs. interorbital space (HW/IOS) Male	2.0-2.2	has part some (head and (bearer of some some (has measurement unit label some space)))) and (has measurement value so
Head width vs. interorbital space (HW/IOS) Male	2.0-2.5	has part some (head and (bearer of some some (has measurement unit label some space)))) and (has measurement value so
Head width vs. interorbital space (HW/IOS) Male	1.8-2.0	has part some (head and (bearer of some some (has measurement unit label some space)))) and (has measurement value so
Intertorular area count	present	has part some intertorular area
Intertorular area count	absent	not (has part some intertorular area)
Lateral margin of harpe shape	widest point of harpe is at its articulation site with gonostyle-volsella complex	(has part some harpe) and (has part som maximum width and (adjacent to some g
Lateral margin of harpe shape	widest point of harpe is in its proximal 1/3rd	(has part some harpe) and (has part som maximum width and (not (adjacent to so
Lateral propodeal carina count	present	has part some lateral propodeal carina
Lateral propodeal carina count	absent	not (has part some lateral propodeal carin
Lateral propodeal carina shape	inverted "U" (left and right lateral propodeal carina are adjacent to the antecostal sulcus of the first abdominal	has part some (lateral propodeal carina a bearer of some median)))) and (has part some antecostal sulcus)))) and (bearer o lateral propodeal carina and (bearer of so

Entity	Value	Semantic
	tergumsubmedially)	
Lateral propodeal carina shape	inverted "V" (left and right lateral propodeal carinae are adjacent medially at their intersection with antecostal sulcus of the first abdominal tergum)	(not (has part some (carina and (bearer lateral propodeal carina and (bearer of so propodeal carina and (bearer of some rig sulcus)
Lateral propodeal carina shape	straight ((left and right lateral propodeal carinae compose a carina that is not broken medially)	(not (has part some (carina and (bearer lateral propodeal carina and (bearer of so propodeal carina and (bearer of some rig
Lateral propodeal carina shape	inverted "Y" (left and right lateral propodeal are adjacent medially posterior to antecostal sulcus of the first abdominal tergum, and connected to the antecostal sulcus by a median carina representing the median branch of the inverted "Y")	(has part some (carina and (has part son lateral propodeal carina and (adjacent to part some (lateral propodeal carina and (some (lateral propodeal carina and (bear
Lateral setae of harpe orientation	oriented distoventrally	has part some (harpe and (has part some sensillum trichodeum and (bearer of som ventral orientation))))))))
Lateral setae of harpe orientation	oriented distally	has part some (harpe and (has part some sensillum trichodeum and (bearer of som
Lateral setae of harpe presence	present	has part some (harpe and (has part some sensillum trichodeum))))
Lateral setae of harpe presence	absent	not (has part some (harpe and (has part sensillum trichodeum)))))
Length of setae on male flagellomere vs. male flagellomere width	setae shorter than width of flagellomeres	has part some (sensillum trichodeum and anatomical line and (bearer of some (len decreased_in_magnitude_relative_to som and (part_of some flagellomere))))))))))
Length of setae on male flagellomere vs. male flagellomere width	setae as long as width of flagellomeres	has part some (sensillum trichodeum and anatomical line and (bearer of some (len similar_in_magnitude_relative_to some (part_of some flagellomere))))))))))

Entity	Value	Semantic
Length of setae on male flagellomere vs. male flagellomere width	setae longer than width of flagellomeres	has part some (sensillum trichodeum and anatomical line and (bearer of some (len increased_in_magnitude_relative_to som and (part_of some flagellomere))))))))))
Male first flagellomere length vs. male second flagellomere length	1.2-1.3	has part some (first flagellomere and (ha line and (bearer of some (length and ((i measurement unit label some (length and anatomical line and (part_of some secon measurement value some float [>= 1.2f ,
Male first flagellomere length vs. male second flagellomere length	1.0-1.1	has part some (first flagellomere and (ha line and (bearer of some (length and ((i measurement unit label some (length and anatomical line and (part_of some secon measurement value some float [>= 1.0f ,
Male first flagellomere length vs. male second flagellomere length	flagellomere 1 is 1.4-1.5 times as long as flagellomere 2	has part some (first flagellomere and (ha line and (bearer of some (length and ((i measurement unit label some (length and anatomical line and (part_of some secon measurement value some float [>= 1.4f ,
Male first flagellomere length vs. male second flagellomere length	1.3-1.4	has part some (first flagellomere and (ha line and (bearer of some (length and ((i measurement unit label some (length and anatomical line and (part_of some secon measurement value some float [>= 1.3f ,
Male first flagellomere length vs. male second flagellomere length	1.1	has part some (first flagellomere and (ha line and (bearer of some (length and ((i measurement unit label some (length and anatomical line and (part_of some secon measurement value some float [>= 1.1f])
Male first flagellomere length vs. male second flagellomere length	1.4	has part some (first flagellomere and (ha line and (bearer of some (length and ((i measurement unit label some (length and anatomical line and (part_of some secon measurement value some float [>= 1.4f])
Male first flagellomere length vs. male second flagellomere length	1.2-1.5	has part some (first flagellomere and (ha line and (bearer of some (length and ((i measurement unit label some (length and anatomical line and (part_of some secon measurement value some float [>= 1.2f ,
Male first flagellomere length vs. male second flagellomere length	1.2-1.4	has part some (first flagellomere and (ha line and (bearer of some (length and ((i

Entity	Value	Semantic
		measurement unit label some (length and anatomical line and (part_of some secon measurement value some float [>= 1.2f ,
Male first flagellomere length vs. male second flagellomere length	1.1-1.2	has part some (first flagellomere and (ha line and (bearer of some (length and ((i measurement unit label some (length and anatomical line and (part_of some secon measurement value some float [>= 1.1f ,
Male first flagellomere length vs. pedicel length	3.2-4.0	has part some (first flagellomere and (ha line and (bearer of some (length and ((i measurement unit label some (length and anatomical line and (part_of some pedice float [>= 3.2f , <= 4.0f])))))))
Male first flagellomere length vs. pedicel length	3.0-3.2	has part some (first flagellomere and (ha line and (bearer of some (length and ((i measurement unit label some (length and anatomical line and (part_of some pedice float [>= 3.0f , <= 3.2f])))))))
Male first flagellomere length vs. pedicel length	1.2-1.3	has part some (first flagellomere and (ha line and (bearer of some (length and ((i measurement unit label some (length and anatomical line and (part_of some pedice float [>= 1.2f , <= 1.3f])))))))
Male first flagellomere length vs. pedicel length	2.5-3.0	has part some (first flagellomere and (ha line and (bearer of some (length and ((i measurement unit label some (length and anatomical line and (part_of some pedice float [>= 2.5f , <= 3.0f])))))))
Male first flagellomere length vs. pedicel length	2.9-3.3	has part some (first flagellomere and (ha line and (bearer of some (length and ((i measurement unit label some (length and anatomical line and (part_of some pedice float [>= 2.9f , <= 3.3f])))))))
Male first flagellomere length vs. pedicel length	2.1-2.4	has part some (first flagellomere and (ha line and (bearer of some (length and ((i measurement unit label some (length and anatomical line and (part_of some pedice float [>= 2.1f , <= 2.4f])))))))
Male first flagellomere length vs. pedicel length	2.5	has part some (first flagellomere and (ha line and (bearer of some (length and ((i measurement unit label some (length and

Entity	Value	Semantic
		anatomical line and (part_of some pedice float [>= 2.5f])))))))
Male first flagellomere length vs. pedicel length	4-4.2	has part some (first flagellomere and (ha line and (bearer of some (length and ((i measurement unit label some (length and anatomical line and (part_of some pedice float [>= 4.0f , <= 4.2f])))))))
Male first flagellomere length vs. pedicel length	2.4-2.5	has part some (first flagellomere and (ha line and (bearer of some (length and ((i measurement unit label some (length and anatomical line and (part_of some pedice float [>= 2.4f , <= 2.5f])))))))
Male ocular ocellar line (OOL):posterior ocellar line (POL):lateral ocellar line(LOL)	2.2:1.1-1.4:1	(has part some (ocular ocellar line and (measured as some (has measurement uni lateral ocellar line)))) and (has measurer has part some (posterior ocellar line and measured as some (has measurement uni lateral ocellar line)))) and (has measurer 1.4f]))))))
Male ocular ocellar line (OOL):posterior ocellar line (POL):lateral ocellar line(LOL)	1.8-2:1.7-1.8:1	(has part some (ocular ocellar line and (measured as some (has measurement uni lateral ocellar line)))) and (has measurer 2.0f])))))) and (has part some (posterior and ((is quality measured as some (has r inheres in some lateral ocellar line)))) an 1.7f , <= 1.8f]))))))
Male ocular ocellar line (OOL):posterior ocellar line (POL):lateral ocellar line(LOL)	2.0-2.1:1.7-1.8:1	(has part some (ocular ocellar line and (measured as some (has measurement uni lateral ocellar line)))) and (has measurer 2.1f])))))) and (has part some (posterior and ((is quality measured as some (has r inheres in some lateral ocellar line)))) an 1.7f , <= 1.8f]))))))
Male ocular ocellar line (OOL):posterior ocellar line (POL):lateral ocellar line(LOL)	1.2-1.3:1:1	(has part some (ocular ocellar line and (measured as some (has measurement uni lateral ocellar line)))) and (has measurer 1.3f])))))) and (has part some (posterior and ((is quality measured as some (has r inheres in some lateral ocellar line)))) an 1.0f]))))))
Male ocular ocellar line (OOL):posterior ocellar line (POL):lateral ocellar line(LOL)	1.4-1.8:1.5-1.8:1	(has part some (ocular ocellar line and (measured as some (has measurement uni

Entity	Value	Semantic
		lateral ocellar line)))) and (has measurem 1.8f])))))) and (has part some (posterior and ((is quality measured as some (has m inheres in some lateral ocellar line)))) an 1.5f , <= 1.8f]))))))
Male ocular ocellar line (OOL):posterior ocellar line (POL):lateral ocellar line(LOL)	1.1-1.2:1.6-1.8:1	(has part some (ocular ocellar line and (measured as some (has measurement uni lateral ocellar line)))) and (has measurem 1.2f])))))) and (has part some (posterior and ((is quality measured as some (has m inheres in some lateral ocellar line)))) an 1.6f , <= 1.8f]))))))
Male ocular ocellar line (OOL):posterior ocellar line (POL):lateral ocellar line(LOL)	1.3-1.5:1:1	(has part some (ocular ocellar line and (measured as some (has measurement uni lateral ocellar line)))) and (has measurem 1.5f])))))) and (has part some (posterior and ((is quality measured as some (has m inheres in some lateral ocellar line)))) an 1.0f]))))))
Male ocular ocellar line (OOL):posterior ocellar line (POL):lateral ocellar line(LOL)	2.9-3.6:2.2-2.1:1	(has part some (ocular ocellar line and (measured as some (has measurement uni lateral ocellar line)))) and (has measurem 3.6f])))))) and (has part some (posterior and ((is quality measured as some (has m inheres in some lateral ocellar line)))) an 2.1f , <= 2.2f]))))))
Male ocular ocellar line (OOL):posterior ocellar line (POL):lateral ocellar line(LOL)	1.1-1.2:1:1	(has part some (ocular ocellar line and (measured as some (has measurement uni lateral ocellar line)))) and (has measurem 1.2f])))))) and (has part some (posterior and ((is quality measured as some (has m inheres in some lateral ocellar line)))) an 1.0f]))))))
Male OOL:LOL	OOL/LOL=1.0-2.0	has part some (ocular ocellar line and (b measured as some (has measurement uni lateral ocellar line)))) and (has measurem 2.0f])))))
Male OOL:LOL	OOL/LOL=0.75-1.0	has part some (ocular ocellar line and (b measured as some (has measurement uni lateral ocellar line)))) and (has measurem 1.0f])))))
Male OOL:POL	OOL/POL=0.5-2.0	has part some (ocular ocellar line and (b

Entity	Value	Semantic
		measured as some (has measurement uni posterior ocellar line)))) and (has measu 2.0f])))))
Male OOL:POL	OOL/POL=0.24-0.43	has part some (ocular ocellar line and (b measured as some (has measurement uni posterior ocellar line)))) and (has measu 0.43f])))))
Mandibular teeth count	2	has part some (mandible and (has compo
Mandibular teeth count	4	has part some (mandible and (has compo
Mandibular teeth count	3	has part some (mandible and (has compo
Mandibular teeth count	1	has part some (mandible and (has compo
Median flange of occipital carina count	absent	not (has part some median flange of occi
Median flange of occipital carina count	present	has part some median flange of occipital
Median mesoscutal sulcus posterior end	adjacent to transscutal articulation	has part some (median mesoscutal sulcus and (adjacent to some transscutal articula
Median mesoscutal sulcus posterior end	not adjacent to transscutal articulation (ends anterior to the articulation)	has part some (median mesoscutal sulcus and (not (adjacent to some transscutal art
Median region of intertorular area shape	flat	has part some (intertorular carina and (h of some flat))))
Median region of intertorular area shape	convex	has part some (intertorular carina and (h of some convex))))
Mesometapleural sulcus count	present dorsally	has part some (mesometapleural sulcus a (has part some ventral region)))))
Mesometapleural sulcus count	present	has part some (mesometapleural sulcus a has part some ventral region))))
Mesometapleural sulcus count	absent	not (has part some mesometapleural sulc
Mesoscutal length vs anterior mesoscutal widthrnrn	MscL/AscW=0.6-0.9	has part some (anteromesoscutum and (l (bearer of some (length and ((is quality label some (length and (inheres in some measurement value some float [>= 0.6f ,

Entity	Value	Semantic
Mesoscutal length vs anterior mesoscutal widthrnrn	MscL/AscW=1.0-2.0	has part some (anteromesoscutum and ([illegible] (bearer of some (length and ((is quality [illegible] label some (length and (inheres in some [illegible] measurement value some float [>= 1.0f , [illegible]
Metapleural carina count	present	has part some metapleural carina
Metapleural carina count	absent	not (has part some metapleural carina)
Notaulus posterior end location	adjacent to transscutal articulation	has part some (notaulus and (has part so[illegible] some transscutal articulation))))
Notaulus posterior end location	anterior to transverse midline of mesoscutum	has part some (mesoscutum and (has pa[illegible] notaulus))))
Notaulus posterior end location	posterior to transverse midline of mesoscutum	has part some (mesoscutum and (has pa[illegible] notaulus))))
Occipital carina sculpture	crenulate	has part some (occipital carina and (bea[illegible]
Occipital carina sculpture	smooth	has part some (occipital carina and (bea[illegible]
Parossiculal seta: number	two	has part some (apical parossiculal seta a[illegible] trichodeum))
Parossiculal seta: number	more than two	has part some (apical parossiculal seta a[illegible] trichodeum))
Parossiculal seta: number	one	has part some (apical parossiculal seta a[illegible] trichodeum))
Parossiculus count (parossiculus and gonostipes fusion)	present (not fused with the gonostipes)	has part some parossiculus
Parossiculus count (parossiculus and gonostipes fusion)	absent (fused with the gonostipes)	not (has part some parossiculus)
Posterior margin of nucha in dorsal view shape	straight	has part some (nucha and (has part som[illegible] straight))))
Posterior margin of nucha in dorsal view shape	concave	has part some (nucha and (has part som[illegible] concave))))
Scutes on posterior region of mesoscutum and dorsal region of mesoscutellum scute convexity	flat	has part some (mesoscutum and (has pa[illegible] some (scute and (has part some dorsal s[illegible]

Entity	Value	Semantic
Scutes on posterior region of mesoscutum and dorsal region of mesoscutellum convexity	convex	has part some (mesoscutum and (has par some (scute and (has part some dorsal su
Postocellar carina	absent	not (has part some postocellar carina)
Postocellar carina	present	has part some postocellar carina
Preoccipital carina and occipital carina structure	occipital carina is short, not extending ventrally to the oral foramen and the preoccipital carina is absent	(not (has part some preoccipital carina)) (not (has part some (ventral region and (
Preoccipital carina and occipital carina structure	the preoccipital carina is absent and the occipital carina extends ventrally to the oral foramen	(not (has part some preoccipital carina)) (adjacent to some oral foramen)))
Preoccipital carina and occipital carina structure	occipital carina complete, preoccipital carina fused laterally with orbital carina	(has part some occipital carina) and (ha preoccipital carina and (adjacent to some
Preoccipital carina and occipital carina structure	the occipital carina is short, interrupted laterally of the occipital foramen, whereas the preoccipital carina extends ventrally to the oral foramen	(has part some (occipital carina and (not and (has part some (preoccipital carina a
Preoccipital carina and occipital carina structure	the occipital carina extends ventrally to the oral foramen with the preoccipital carina present on the vertex, but not extendinig ventrally along the gena	(has part some (occipital carina and (ha to some oral foramen))))) and (has part s preoccipital carina and (not (has part son
Preoccipital carina and occipital carina structure	the preoccipital carina is absent from the vertex, but a carina, that is continuous with the anterior margin of the preoccipital lunula extends ventrally to the oral foramen	(has part some (preoccipital lunula and (part some (carina and (adjacent to some vertex and (not (has part some preoccipit
Preoccipital carina count	absent	not (has part some preoccipital carina)

Entity	Value	Semantic
Preoccipital carina count	present	has part some preoccipital carina
Preoccipital carina shape	present medially, absent laterally to lateral ocelli	has part some (preoccipital carina and (h some (medial region and (not (has part s
Preoccipital carina shape	complete	has part some (preoccipital carina and (h some (medial region and (has part some
Preoccipital carina shape	interrupted dorsally and represented by irregular, not continuous carinae	has part some (preoccipital carina and (h some medial region) and (has part some inconspicuous))))
Preoccipital furrow anterior end	Preoccipital furrow ends inside ocellar triangle	has part some (preoccipital furrow and (anterior_to some posterior ocellar line)))
Preoccipital furrow anterior end	Preoccipital furrow ends prosterior to ocellar triangle	has part some (preoccipital furrow and (posterior_to some interocellar space))))
Preoccipital furrow count	present	has part some preoccipital furrow
Preoccipital furrow count	absent	not (has part some preoccipital furrow)
Preoccipital lunula count	present	has part some preoccipital lunula
Preoccipital lunula count	absent	not (has part some preoccipital lunula)
Presence of intertorular carina	present	has part some intertorular carina
Presence of intertorular carina	absent or reduced medially	has part some (intertorular carina and (no
Proximodorsal notch of cupula count	present	has part some proximodorsal notch of cup
Proximodorsal notch of cupula count	absent	not (has part some proximodorsal notch o
Proximodorsal notch of cupula shape	notched	has part some (proximodorsal notch of cu
Proximodorsal notch of cupula shape	arched	has part some (proximodorsal notch of cu
Proximodorsal notch of cupula width vs length	at least two times as long as wide	has part some (anatomical line and (bear measured as some (has measurement uni anatomical line)))) and (has measuremen
Proximodorsal notch of cupula width vs length	wider than long	has part some (proximodorsal notch of cu anatomical line and (bearer of some (len decreased_in_magnitude_relative_to som

Entity	Value	Semantic
		and (part_of some proximodorsal notch c
Proximodorsal notch of cupula width vs length	as long as wide	has part some (proximodorsal notch of cu anatomical line and (bearer of some (len similar_in_magnitude_relative_to some (part_of some proximodorsal notch of cup
Proximolateral corner of male S9 shape	blunt	has part some (abdominal sternum 9 and some (proximal side and ((bearer of som blunt)))))))
Proximolateral corner of male S9 shape	acute	has part some (abdominal sternum 9 and some (proximal side and ((bearer of som acute)))))))
Proximolateral projection of the cupula shape	blunt	has part some (proximolateral projection
Proximolateral projection of the cupula shape	acute	has part some (proximolateral projection
Randomly sized areolae around setal pits on frons count	absent	has part some (frons and (has part some pit)))))
Randomly sized areolae around setal pits on frons count	present	has part some (frons and (has part some
Rugose region on frons count	present	has part some (frons and (has part some rugose))))
Rugose region on frons count	absent	not (has part some (frons and (has part s some rugose)))))
S1 length vs. shortest width	S1 wider than long	has part some (anterior area of the secon median anatomical line and (bearer of so some (has measurement unit label some and (has measurement value some float [
S1 length vs. shortest width	S1 longer than wide	has part some (anterior area of the secon median anatomical line and (bearer of so some (has measurement unit label some and (has measurement value some float [
Scutoscutellar sulcus vs transscutal articulation	adjacent	has part some (scutoscutellar sulcus and articulation))
Scutoscutellar sulcus vs transscutal articulation	not adjacent	has part some (scutoscutellar sulcus and articulation)))

Entity	Value	Semantic
Sensillar patch of the male flagellomere pattern	F6-F9	(has part some (sixth flagellomere and ([illegible] flagellomere))) and (has part some (sev[illegible] sensillar patch of the male flagellomere)[illegible] and (has part some sensillar patch of the [illegible] ninth flagellomere and (has part some se[illegible]
Sensillar patch of the male flagellomere pattern	F4-F9	(has part some (fifth flagellomere and ([illegible] flagellomere))) and (has part some (fou[illegible] sensillar patch of the male flagellomere)[illegible] and (has part some sensillar patch of the [illegible] seventh flagellomere and (has part some [illegible] and (has part some (eighth flagellomere [illegible] male flagellomere))) and (has part some [illegible] sensillar patch of the male flagellomere)[illegible]
Sensillar patch of the male flagellomere pattern	F5-F9	(has part some (fifth flagellomere and ([illegible] flagellomere))) and (has part some (sixt[illegible] sensillar patch of the male flagellomere)[illegible] flagellomere and (has part some sensillar [illegible] has part some (eighth flagellomere and ([illegible] flagellomere))) and (has part some (nin[illegible] sensillar patch of the male flagellomere)[illegible]
Sensillar ring area of harpe orientation	medially	has part some (area and (surrounded by [illegible] of some medial orientation))))
Sensillar ring area of harpe orientation	dorsomedially	has part some (area and (surrounded by [illegible] of some (dorsal orientation and (bearer [illegible]
Setal pit on vertex size	larger than diameter of scutes	has part some (setal pit and (has part so[illegible] (length and (increased_in_magnitude_re[illegible] some (maximum width and (part_of som[illegible]
Setal pit on vertex size	smaller than diameter of scutes	has part some (setal pit and (has part so[illegible] (length and (decreased_in_magnitude_re[illegible] some (maximum width and (part_of som[illegible]
Speculum ventral limit	not extending ventrally of pleural pit line	not (has part some (speculum and (has [illegible] posterior_to some (posterior margin and [illegible]
Speculum ventral limit	extending ventrally of pleural pit line	has part some (speculum and (has part s[illegible] some (posterior margin and (part_of som[illegible]
Sternaulus count	absent	not (has part some sternaulus)
Sternaulus count	present	has part some sternaulus
Sternaulus length	short, not reaching 1/2 of	has part some (sternaulus length and (be[illegible]

Entity	Value	Semantic
	mesopleuron length at level of sternaulus	measured as some (has measurement uni mesopleuron length)))) and (has measur
Sternaulus length	elongate, exceeding 3/4 of mesopleuron length at level of sternaulus	has part some (sternaulus length and (be measured as some (has measurement uni mesopleuron length)))) and (has measur
Submedian flanges of occipital carina count	present	has part some submedial flange of occipit
Submedian flanges of occipital carina count	absent	not (has part some submedial flange of o
Subtorular carina count	present	has part some subtorular carina
Subtorular carina count	absent	not (has part some subtorular carina)
Supraclypeal depression count	absent	not (has part some supraclypeal depressi
Supraclypeal depression count	present	has part some supraclypeal depression
Supraclypeal depression structure	absent medially, represented by two grooves laterally of facial pit	has part some (supraclypeal depression a
Supraclypeal depression structure	present medially, inverted U-shaped	has part some (supraclypeal depression a
Torulo-clypeal carina count	present	has part some torulo-clypeal carina
Torulo-clypeal carina count	absent	not (has part some torulo-clypeal carina)
Transverse carina on petiole shape	concave	has part some (transverse carina on petio
Transverse carina on petiole shape	straight	has part some (transverse carina on petio
Transverse frontal carina count	present	has part some transverse frontal carina
Transverse frontal carina count	absent	not (has part some transverse frontal cari
Transverse line of the metanotum/propodeum fusion with antecosta of abdominal tergum 1.	fused medially	has part some (transverse line of the meta (antecostal sulcus of the first abdominal region)) and (bearer of some medial reg

Entity	Value	Semantic
Transverse line of the metanotum/propodeum fusion with antecosta of abdominal tergum 1.	fused sublaterally	has part some (transverse line of the meta (antecostal sulcus of the first abdominal region)) and (bearer of some lateral regi
Transversely reticulate region on frons count	present	has part some (frons and (has part some reticulate and (bearer of some horizontal
Transversely reticulate region on frons count	absent	not (has part some (frons and (has part some (reticulate and (bearer of some hor
Transversely reticulate region on frons extent	extending entire width of frons	has part some (frons and (has part some reticulate and (bearer of some horizontal
Transversely reticulate region on frons extent	restricted to lateral branches of supraclypeal depression	has part some (supraclypeal depression a has part some (anatomical region and (b some horizontal))))))))
Ventral margin of torulus vs dorsal margin of clypeus	adjacent	has part some (torulus and (has part som dorsal margin and (part_of some clypeus
Ventral margin of torulus vs dorsal margin of clypeus	not adjacent	has part some (torulus and (has part som some (dorsal margin and (part_of some
Ventrolateral invagination of the pronotum presence	absent	not (has part some ventrolateral invagina
Ventrolateral invagination of the pronotum presence	present	has part some ventrolateral invagination
Ventromedian and ventrolateral white, setfierous patches on frons count	present	(has part some (ventromedian setiferous has part some (ventrolateral setiferous pa
Ventromedian and ventrolateral white, setfierous patches on frons count	absent	not ((has part some (ventromedian setife and (has part some (ventrolateral setifer
Weber length	WL=3.0-4.5	(has part some Weber length) and (bear measured as some (has measurement uni measurement value some (float [>= 400.
Whitish, thick setae on frons count	absent	has part some (frons and (not (has part s of some white)))))
Whitish, thick setae on frons count	present	has part some (frons and (has part some some white))))
